# *PpHYH* is responsible for light-induced anthocyanin accumulation in fruit peel of *Prunus persica*

**DOI:** 10.1093/treephys/tpac025

**Published:** 2022-02-26

**Authors:** Lei Zhao, Juanli Sun, Yaming Cai, Qiurui Yang, Yuanqiang Zhang, Collins Otieno Ogutu, Jingjing Liu, Yun Zhao, Furong Wang, Huaping He, Beibei Zheng, Yuepeng Han

**Affiliations:** CAS Key Laboratory of Plant Germplasm Enhancement and Specialty Agriculture, The Innovative Academy of Seed Design of Chinese Academy of Sciences, Wuhan Botanical Garden, Wuhan 430074, China; University of Chinese Academy of Sciences, 19A Yuquanlu, Beijing 100049, China; CAS Key Laboratory of Plant Germplasm Enhancement and Specialty Agriculture, The Innovative Academy of Seed Design of Chinese Academy of Sciences, Wuhan Botanical Garden, Wuhan 430074, China; University of Chinese Academy of Sciences, 19A Yuquanlu, Beijing 100049, China; CAS Key Laboratory of Plant Germplasm Enhancement and Specialty Agriculture, The Innovative Academy of Seed Design of Chinese Academy of Sciences, Wuhan Botanical Garden, Wuhan 430074, China; University of Chinese Academy of Sciences, 19A Yuquanlu, Beijing 100049, China; CAS Key Laboratory of Plant Germplasm Enhancement and Specialty Agriculture, The Innovative Academy of Seed Design of Chinese Academy of Sciences, Wuhan Botanical Garden, Wuhan 430074, China; University of Chinese Academy of Sciences, 19A Yuquanlu, Beijing 100049, China; CAS Key Laboratory of Plant Germplasm Enhancement and Specialty Agriculture, The Innovative Academy of Seed Design of Chinese Academy of Sciences, Wuhan Botanical Garden, Wuhan 430074, China; University of Chinese Academy of Sciences, 19A Yuquanlu, Beijing 100049, China; CAS Key Laboratory of Plant Germplasm Enhancement and Specialty Agriculture, The Innovative Academy of Seed Design of Chinese Academy of Sciences, Wuhan Botanical Garden, Wuhan 430074, China; CAS Key Laboratory of Plant Germplasm Enhancement and Specialty Agriculture, The Innovative Academy of Seed Design of Chinese Academy of Sciences, Wuhan Botanical Garden, Wuhan 430074, China; University of Chinese Academy of Sciences, 19A Yuquanlu, Beijing 100049, China; CAS Key Laboratory of Plant Germplasm Enhancement and Specialty Agriculture, The Innovative Academy of Seed Design of Chinese Academy of Sciences, Wuhan Botanical Garden, Wuhan 430074, China; Hubei Hongshan Laboratory, Wuhan, Hubei 430070, China; Institute of Fruit Tree and Tea, Academy of Agricultural Science, Wuhan, Hubei 430209, China; Institute of Fruit Tree and Tea, Academy of Agricultural Science, Wuhan, Hubei 430209, China; CAS Key Laboratory of Plant Germplasm Enhancement and Specialty Agriculture, The Innovative Academy of Seed Design of Chinese Academy of Sciences, Wuhan Botanical Garden, Wuhan 430074, China; Hubei Hongshan Laboratory, Wuhan, Hubei 430070, China; CAS Key Laboratory of Plant Germplasm Enhancement and Specialty Agriculture, The Innovative Academy of Seed Design of Chinese Academy of Sciences, Wuhan Botanical Garden, Wuhan 430074, China; Hubei Hongshan Laboratory, Wuhan, Hubei 430070, China

**Keywords:** anthocyanin, light, *PpCOP1*, *PpHYH*, *Prunus persica*

## Abstract

Peach *Prunus persica* is an economically important fruit tree crop worldwide. Although the external color of fruit is an important aspect of fruit quality, the mechanisms underlying its formation remain elusive in peach. Here, we report an elongated hypocotyl 5-homolog gene *PpHYH* involved in the regulation of anthocyanin pigmentation in peach fruit peel. Anthocyanin accumulation in fruit peel is light-dependent in peach. *PpHYH* had no auto-activation activity and its transcription was induced by sunlight. *PpHYH* activated transcription of a cluster of three *PpMYB10* genes in the present of a cofactor *PpBBX4* encoding a B-BOX protein, leading to anthocyanin accumulation in the sun-exposed peel. However, the *PpHYH* activity was repressed by a negative regulator of *PpCOP1* encoding constitutive photomorphogenesis protein 1 which accumulated in the nucleus under dark condition, resulting in failure of anthocyanin accumulation in the shaded peel. PpCOP1 was re-localized into the cytosol under light condition, in accordance with fruit peel pigmentation. Additionally, transport of anthocyanins from the cytoplasm to the vacuole was a rate-limiting step for anthocyanin accumulation in peach fruit peel. Our results reveal for the first time the *HYH* gene involved in the regulation of anthocyanin accumulation in fruits, and provide target genes for genetic manipulation of fruit coloration.

## Introduction

Plant coloration is mainly attributed to the accumulation of pigments, including chlorophylls, carotenoids, betaines and flavonoids. Anthocyanins are an important subgroup of flavonoids, providing a wide variety of colors in most fruits and vegetables, ranging from the bright red-orange to blue-violet colors (Sass-Kiss et al. 2005, Lightbourn et al. 2008). The accumulation of anthocyanins can resist the damage caused by excessive light in plants (Winkel 2002). Notably, anthocyanins are antioxidants that may provide a range of health benefits, from inhibiting the aging process to preventing cardiovascular diseases (He and Giusti 2010). Hence, anthocyanins have attracted much attention in the breeding programs of fruits and vegetables.

Genetic study of anthocyanin synthesis, one of the best-studied metabolic pathways in plants, can be traced back to Mendel’s work on flower color of peas in the early 20th century (Weldon 1901, Holton and Cornish 1995). Anthocyanins are synthesized via the phenylpropanoid pathway, which involves a number of enzymes, such as chalcone synthase (CHS), chalcone isomerase (CHI), flavanone 3-hydroxylase (F3H), flavonoid 3′-hydroxylase (F3′H), dihydroflavone alcohol-4-reductase (DFR), leucoanthocyanidin dioxygenase (LDOX) and UDP glucose: flavonoid-3-*O*-glucosyltransferase (UFGT). Anthocyanin biosynthesis occurs mainly in the endoplasmic reticulum (ER) and is regulated at the transcriptional level by the MYB-bHLH-WD40 (MBW) complex containing MYB and bHLH transcription factors (TFs) as well as a WD40 repeat protein (Petroni and Tonelli 2011, Xu et al. 2015). Anthocyanins are only stable under highly acidic conditions and can be degraded quickly under neutral conditions (Cabrita et al. 2014). Therefore, following the ir synthesis in the cytoplasm, anthocyanins are transported to the vacuole where they are stored in the acidic environment. Transport of anthocyanins from ER to the vacuole are mediated by at least two types of transporters, multidrug and toxic compound extrusion (MATE) and glutathione-S-transferase (GST) (Shitan and Yazaki 2013). Loss-of-function mutations in the *TT12* and *TT19* genes encoding MATE and GST, respectively, both cause failure of anthocyanin accumulation in *Arabidopsis* (Marinova et al. 2007, Sun et al. 2012).

Light is an important regulator of various biological processes such as anthocyanin accumulation in plants (Jiao et al. 2007). Under dark conditions, constitutive photomorphogenesis protein 1 (COP1), a central regulator of light signaling, accumulates in the nucleus to mediate the ubiquitin-dependent degradation of various proteins, including anthocyanin-related regulators such as elongated hypocotyl 5 (HY5) and MYB TFs production of anthocyanin pigments 1/2 (PAP1/PAP2) (Osterlund et al. 2000, Saijo et al. 2003, Maier et al. 2013). After exposure to light, COP1 translocates from the nucleus into the cytoplasm (Jiao et al. 2007, Lau and Deng 2012). The photoreceptors such as phytochromes, cryptochromes (CRYs) and UV Resistance Locus 8 (UVR8) suppress COP1 activity, leading to the stabilization of the COP1 substrates (Vonarnim and Deng 1994, Maier et al. 2013). The regulatory roles of light in anthocyanin coloration have been reported in fruit crops such as apple, pear and strawberry, and both *HY5* and *COP1* genes have been shown to be crucial for light-induced anthocyanin accumulation (Takos et al. 2006, Li et al. 2012, Bai et al. 2019, Li et al. 2020). Although HY5 shows an ability to activate its own promoter in vivo (Abbas et al. 2014), it actually has no transcriptional activation domain and requires cofactors to activate target gene expression (Stracke et al. 2010). A recent study reveals B-box-containing proteins (BBXs) as essential partners for HY5-dependent modulation of anthocyanin accumulation (Bursch et al. 2020). Apart from HY5, elongated hypocotyl 5-homolog (HYH) has also been found to participate in anthocyanin accumulation in *Arabidopsis* (Zhang et al. 2011).

Peach (*Prunus persica*) is an economically important fruit crop worldwide. Anthocyanin accumulation in peach fruit is mainly regulated by a cluster of three R2R3-MYB genes, *PpMYB10.1*, *PpMYB10.2* and *PpMYB10.3*, which have functionally diverged (Rahim et al. 2014, Zhou et al. 2018). *PpMYB10.1* is involved in the regulation of anthocyanin accumulation in exocarp and mesocarp and its transcription can be activated by a heterodimer formed by two NAM/ATAF/CUC (NAC) TFs, PpBL and PpNAC1 (Zhou et al. 2015). Anthocyanin pigmentation in peach leaf is controlled by an additional R2R3-MYB gene *PpMYB10.4* in the *Gr* locus (Zhou et al. 2014). Besides *PpMYB10* TFs, a *PpGST* gene (also known as *Riant*) has also proven to play an important role in anthocyanin coloration of both flowers and fruits (Cheng et al. 2015, Zhao et al. 2020, Lu et al. 2021). In addition, a recent study shows that treatments with both Ultraviolet A (320–420 nm) and Ultraviolet B (275–320 nm) promote fruit skin pigmentation by activating the expression of a *PpHY5* gene in peach (Zhao et al. 2021). However, molecular mechanisms of the light-induced anthocyanin accumulation remain largely unclear in peach.

In this study, we investigated mechanisms underlying the impact of sunlight on anthocyanin accumulation in fruit peel of *Prunus persica*. A *PpHYH* gene was found to be a light-response regulator controlling anthocyanin accumulation in fruit peel under light condition. Light triggers the export of PpCOP1, a negative regulator of PpHYH, from the nucleus to the cytoplasm, which makes it possible that PpHYH interacts with its cofactor PpBBX4 to activate transcription of *PpMYB10* genes. Our results will be helpful for gaining a comprehensive understanding of the complex mechanisms underlying anthocyanin accumulation in peach fruit.

## Materials and methods

### Plant materials

Two peach cultivars ‘MLWN’ and ‘XHJ’ as well as a nectarine cultivar ‘YGYT’ used in this study are maintained in Wuhan Botanical Garden of Chinese Academy of Sciences, Wuhan, China. Fruit samples of ‘MLWN’ and ‘YGYT’ were collected 45 days after full bloom (DAF), with a clear difference in color between sun-exposed and shaded sides. The peel from either the sun-exposed or shaded sides of the fruit was collected. The samples were immediately frozen in liquid nitrogen and then stored at −75 °C until use. Each cultivar consisted of three biological replicates, with each containing at least three fruits. Tobacco seedlings (*Nicotiana benthamiana*) used in this study were grown in a growth room set at 25 °C and a daylight cycle of 16–8 h. Transient expression assay was conducted using fruit samples at early ripening stages collected from ‘XHJ’. Additionally, strawberry cultivar ‘Hongyan’ grown in the greenhouse with natural light and temperature of 16–28°C was kindly provided by Institute of Economic Crops, Hubei Academy of Agricultural Sciences, Wuhan, China.

### Extraction and quantification of anthocyanins and flavonols

Total anthocyanin was measured by the pH differential method with minor modifications (Fuleki and Francis 1968). Approximately 100 mg sample was ground into powder, transferred into a 1.5-ml centrifuge tube and mixed with 1 ml of 0.1% hydrochloric acid methanol. After incubation at 4 °C for 20 h, the mixture was centrifuged with 13,400*g* at room temperature for 20 min. The supernatants were transferred to a 5-ml centrifuge tube and diluted with 0.1% hydrochloric acid methanol to a final volume of 3 ml. Two 500 μl aliquots of a sample were individually transferred into a 2-ml centrifuge tube and then diluted with Buffer A (0.2 M KCl/0.2 M HCl, 25:67, v/v, pH 1.0) and Buffer B (0.2 M NaAc, pH 4.5), respectively. After incubation at 4 °C for 2 h, the absorbance of the diluted samples at pH 1.0 and pH 4.5 was measured at a wavelength of 510 and 700 nm using a multifunctional microplate reader (TECAN Infinite M200, Austria). Then, the absorbance was calculated based on the formula as follows: Abs = [(A510–A700) pH 1.0−(A510–A700) pH 4.5]. Anthocyanin concentration was estimated using the following equation: Abs/g fresh weight (FW). Three biological replicates were performed for each sample.

The content of total flavonols was determined using the colorimetric method with minor modifications (Amoussa et al. 2015). Approximately 200 mg sample was ground into powder, transferred into a 1.5-ml centrifuge tube and mixed with 0.5 ml of 1% formic acid methanol. After 30 min ultrasonic treatment, the mixture was centrifuged at 13,400*g* for 10 min at room temperature. The supernatants were transferred to a 2-ml microcentrifuge tube and the pellet was extracted twice using the same protocol described above, and the supernatants were combined. Approximately 500 μl of supernatant was mixed with 500 μl of 20% AlCl_3_. The mixture was incubated at room temperature for 10 min. Fluorescence was measured at a wavelength of 425 nm using multifunctional microplate reader. The concentration of flavonols was calculated using the following equation: Abs/g FW. Three biological replicates were performed for each sample.

### RNA extraction and quantitative real-time polymerase chain reaction analysis

Total RNA was extracted using the EASYspin Plus Plant RNA rapid Extraction Kit (RN38, Aidlab, Beijing, China). RNA concentration was measured using NanoDrop lite ultraviolet spectrophotometer (ND-LITE-PR, USA) and all samples were diluted using ultra-pure water to a final concentration of 500 ng μl^−1^. The first-strand cDNA was synthesized using PrimerScript™RT reagent Kit with gDNA Eraser (Takara, Dalian, China). Quantitative real-time polymerase chain reaction (qRT-PCR) was conducted in a total reaction volume of 20 μl containing 0.2 μl of each primer, 10 μl of HieffTM qPCR SYBR® Green Master Mix (Yeasen, Shanghai, China), and 2 μl of cDNA template. The program of qRT-PCR was as follows: one cycle of 1 min at 95 °C, followed by 40 cycles of 10 s at 95°C and 30 s at 60 °C. Polymerase chain reaction amplification was performed using the StepOnePlus™ Real-Time PCR System (7300 Applied Biosystems™, USA). A previously reported gene *GADPH* in peach was used as an internal control (Tong et al. 2009). The relative gene expression levels were calculated following the cycle threshold (Ct) 2^–ΔΔCt^ method. Three biological replicates were performed for each sample. The sequences of primers used for qRT-PCR are listed in Table S1 available as Supplementary data at *Tree Physiology* Online.

### RNA-seq library construction, sequencing and data analysis

Total RNA was extracted using Trizol reagent according to our previous report (Zhou et al. 2015). Approximately 20 mg of total mRNA was enriched using the oligo (dT) magnetic beads. After adding the fragmentation buffer, the mRNA was interrupted to short fragments. The mRNA fragments were used as templates to synthesize the first-strand cDNA using random hexamer-primers and dNTPs, RNase H, buffer and DNA polymerase I were subsequently added to synthesize the second strand. Double-strand cDNA was purified using AMPure XP beads and then subjected to end repair, A-tailing, and sequencing adaptor ligation using the KAPA Hyper Prep Kit (Kapa Biosystems, MA, USA). Polymerase chain reaction amplification for the fragment enrichment was performed using qPCR BIO-RAD CFX96 system (Bio-Rad, USA) to generate library products. Library quality was ensured by accurate quantification of the effective concentration of the library (effective library concentration >2 nM). The insert size was estimated using the Agilent 2100 bioanalyzer (Agilent, Santa Clara, CA, USA). Sequencing of library products was carried out using an Illumina HiSeq Xtenq platform. Each treatment consisted of three biological replicates.

Raw data were trimmed by removing adaptor sequences, empty reads and low-quality sequences and then mapped to the reference genome of peach (Verde et al. 2013) using HISAT2 and Bowtie 2 (Langmead and Salzberg 2012). Pearson’s correlation coefficient and principal component analysis were used to estimate the consistency of biological replicates. Gene expression levels were calculated based on expected number of fragments per kilobase of transcript sequence per millions base pairs sequenced. Differentially expressed genes (DEGs) were identified using the program DESeq2 (Love et al. 2014), with the following parameters: fold change ≥2 and false discovery rate ≤0.05.

### Phylogenetic analysis and proteins sequence alignment

Amino acid sequences of the *PpHYH* gene and its homologs in other fruit crops and the model plant *Arabidopsis thaliana* were retrieved from National Center for Biotechnology Information (NCBI). Amino acid sequences were aligned using CLUSTALX and the resulting data matrix was used to construct phylogenetic tree with the neighbor-joining method using the MEGA6 software.

### Subcellular localization assay

The cDNA templates prepared from fruits of ‘MLWN’ were used to amplify the entire coding DNA sequences of the *PpHYH* gene. The cDNA fragment was inserted into the BamHI-digested vector pFGC-YFP with the homologous recombinant method using the pEASY-Basic Seamless Cloning and Assembly Kit (TransGen Biotech, Beijing, China). The expression vector was transformed into the *Agrobacterium tumefaciens* strain GV3101 using the heat shock method and incubated at 28 °C for 2 days. The bacterium was resuspended in 10 ml of infiltration buffer containing 20 μM acetosyringone, 10 mM 2-(*N*-morpholine)-ethanesulfonic acid (pH 5.7) and 10 mM MgCl_2_, and incubated without shaking at 25 °C for 2 h. *Agrobacterium* cultures were injected into young leaves of 3-week-old tobacco seedlings. Three days after infiltration, fluorescence was measured using the confocal microscope (TCS SP8, Leica, Microsystems, Wetzlar, Germany). DNA sequences of the primers used for vector construction are listed in Table S2 available as Supplementary data at *Tree Physiology* Online.

### Yeast one-hybrid and yeast two-hybrid assays

The full-length coding sequence of the *PpHYH* gene was amplified from the cDNA templates of fruits of ‘MLWN’ and inserted individually into the pGBKT7 vector. The expression vector was transferred into yeast strain yeast two-hybrid (Y2H) Gold. To verify self-activation activity of the fusion construct, transformant was incubated on SD-Trp and SD-Trp/X-A-Gal/AbA plates for 3 days, respectively. Then, the construct of DNA-binding domain (BD) bait was used to screen an AD-cDNA library (the Gal4 activation domain fused to peach fruit cDNA fragments) that was developed from the cDNA templates synthesized from peach fruits at different developmental stages. Positive colonies were sequenced and their corresponding sequences were individually inserted into the pGADT7 vector. The BD–bait and the AD–prey constructs were transformed into yeast strain Y2H gold and incubated on DDO (SD−Trp/Leu), QDO (SD−Trp/Leu/His/Ade) and QDO/X/A (SD−Trp/Leu/His/Ade/X—A-gal/AbA) plates for at least 3 days. Y2H assay was conducted using the Matchmaker™ Gold Yeast two-hybrid System (Clontech, http://www.clontech.com/).

For yeast one-hybrid (Y1H), the full-length coding sequence of the *PpHYH* gene was inserted into the pGADT7 vector. Promoter sequences of *PpMYB10.1* and *PpHYH*, approximately 1.5 and 2.0 kb, respectively, upstream of the start codon, were amplified from ‘MLWN’ and inserted individually into the reporter vector pAbAi. The pAbAi bait constructs were digested with *Bst*BI (New England Biolabs, Beverly, MA, USA) and subsequently transferred into yeast strain Y1H Gold. The transformed yeast strains were selected on SD medium lacking uracil to determine the minimal inhibitory concentration of Aureobasidin A (AbA) for the pAbAi bait constructs. The pGADT7-PpHYH construct was then transferred into the transformed Y1H yeast containing the promoter regions of either *PpMYB10.1* or *PpHYH*. The interactions of the pGADT7-PpHYH construct with the pAbAi bait constructs were estimated based on positive yeast cells grown on SD/-Ura/AbA^*^ medium (^*^ means the minimum concentration of AbA mentioned above). DNA sequences of the primers used for vector construction are listed in Table S2 available as Supplementary data at *Tree Physiology* Online.

### Firefly luciferase complementation assay

Firefly luciferase complementation assay was carried out following to our previous study (Zhou et al. 2015). The full-length coding sequence of *PpHYH* was inserted into binary vector pCambia1300-cLUC, while whole coding sequence of *PpBBX4* without the stop codon was inserted into binary vector pCambia1300-nLUC. The resulting constructs were individually transformed into the *A. tumefaciens* strain GV3101. *Agrobacterium* culture and infiltration preparation were performed following the same protocol as described for subcellular localization assay.

After 2 days of infiltration, fluorescent photographs were taken to display firefly luciferase activity using an ImageQuant LAS4000 mini chemiluminescence imaging system (GE Life Sciences). At least three biological replicates were performed for each assay. DNA sequences of primers used for vector construction are listed in Table S2 available as Supplementary data at *Tree Physiology* Online.

### Dual luciferase reporter assay

The promoter regions of *PpMYB10.1* and *PpHYH*, ~1.5 and 2.0 kb, respectively, upstream of the start codon, were inserted into the pGreenII 0800-LUC vector. The full-length coding sequences of *PpHYH* and *PpBBX4* were inserted into the vector pSAK277 and the *GUS* gene was used as a negative control (Zhou et al. 2015). All the resulting constructs were individually transformed into the *A. tumefaciens* strain GV3101. *Agrobacterium* cultivation and infiltration preparation were performed according to the same protocol as described for subcellular localization assay. After 3 days of infiltration, leaf disks with 2 cm in diameter that were adjacent to the infiltration site were punched to measure the firefly and ranilla luciferase fluorescence intensity using Dual-Luciferase Reporter Gene Assay Kit (Promega). The firefly and ranilla luciferase fluorescence activity was expressed in the Luc/Ren ratio. At least three biological replicates were performed for each treatment. DNA sequences of primers used for vector construction are listed in Table S2 available as Supplementary data at *Tree Physiology* Online.

### Functional analysis of genes through their transient expression in peach and strawberry fruits

Whole coding sequences of *PpHYH* and *PpBBX4* were individually inserted into the pSAK277 vector and then transformed into the *A. tumefaciens* strain GV3101 using the above- mentioned protocols. *Agrobacterium* cultivation and infiltration preparation were performed following the same protocol as described for subcellular localization assay. For peach, fruits after *Agrobacterium* infiltration were placed in a growth chamber at 25 °C under 16-h light/8-h light photoperiod. Photos were taken 5 days after infiltration and fruit samples around the infiltration sites were collected, frozen with liquid nitrogen and then stored at −75 °C until use. For strawberry, the plants after *Agrobacterium* infiltration were placed in the greenhouse with natural light and temperature of 10–26 °C. Fruit samples were taken 7 days after infiltration. DNA sequences of the primers used for expression vector construction are listed in Table S2 available as Supplementary data at *Tree Physiology* Online.

## Results

### Light showed a considerable influence on anthocyanin accumulation in the exocarp of peach fruits

**Figure 1.**
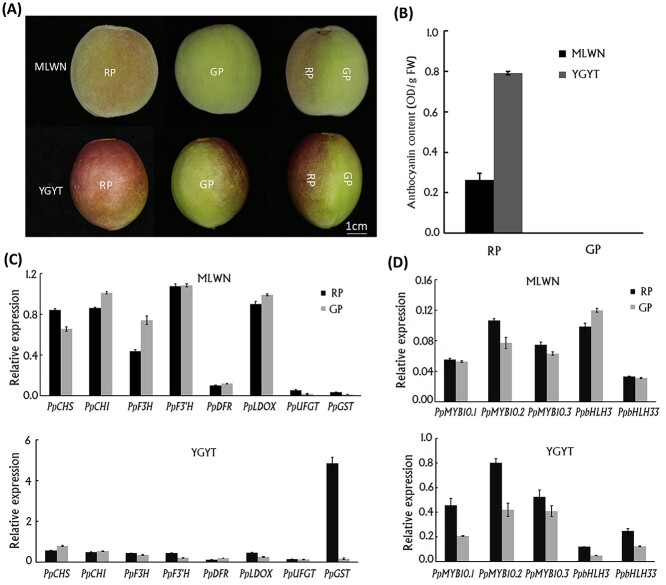
Effect of light on anthocyanin accumulation in the fruit exocarp of peach cv. MLWN and YGYT. (A) The difference in coloration between sun-exposed (left) and shaded (middle) sides of: (B) anthocyanin accumulation in the sun-exposed and shaded peel tissues; (C) expression of anthocyanin structural genes in the sun-exposed and shaded peel tissues; and (D) expression of anthocyanin regulatory genes in the sun-exposed and shaded peel tissues. RP, the sun-exposed red peel; GP, the shaded green peel.

A peach cv. ‘MLWN’ and a nectarine cv. ‘YGYT’ were selected to investigate the effect of light on anthocyanin accumulation in the exocarp. Fruits of ‘MLWN’ at 45 DAF showed a clear difference in coloration between sun-exposed (red) and shaded (green) sides (Figure 1A). For ease of description, the sun-exposed and shaded sides were designated red peel (RP) and green peel (GP), respectively. Consistent with the difference in fruit skin coloration, anthocyanin accumulation was detected in the sun-exposed side, while not detectable in the shaded side (Figure 1B). Likewise, fruits of ‘YGYT’ at 45 DAF displayed red and green colors in the sun-exposed and shaded sides, respectively (Figure 1A). Anthocyanin accumulation occurred only in the sun-exposed side (Figure 1B). These results indicated that light was indispensable for anthocyanin accumulation in the fruit exocarp of *P. persica*.

### Effect of light on the transcription of anthocyanin structural and regulatory genes

The expression levels of anthocyanin structural and regulatory genes were investigated in sun-exposed and shaded sides of ‘MLWN’ and ‘YGYT’. For ‘MLWN’, anthocyanin pathway genes, *PpCHS*, *PpUFGT* and *PpGST*, showed higher levels of expression in the sun-exposed side than in the shaded side (Figure 1C). However, it was unexpected that the majority of anthocyanin biosynthetic genes, such as *PpCHI*, *PpF3H*, *PpF3′H*, *PpDFR* and *PpLDOX*, had higher levels of expression in the shaded side than in the sun-exposed side. For ‘YGYT’, anthocyanin pathway genes, such as *PpF3H*, *Pp3′H*, *PpLDOX* and *PpGST*, had higher levels of expression in the sun-exposed side than in the shaded side (Figure 1C), while the opposite result was observed for *PpCHS*, *PpCHI* and *PpDFR.* The *PpUFGT* gene showed no difference in expression levels between the sun-exposed and shaded sides. These results indicated that anthocyanin transport gene *PpGST* rather than anthocyanin biosynthetic genes showed a consistent activation in the sun-exposed peel of both peach and nectarine cultivars.

The expression levels of anthocyanin-activating *R2R3-MYB* genes, *PpMYB10.1*, *PpMYB10.2* and *PpMYB10.3*, were higher in the sun-exposed peel than in the shaded peel of both ‘MLWN’ and ‘YGYT’ (Figure 1D). Similar result was also observed for *PpbHLH33*. However, *PpbHLH3* showed lower levels of expression in the sun-exposed peel than in the shaded peel in ‘MLWN’, but the opposite result was detected in ‘YGYT’. These results indicated the important roles of anthocyanin-activating *R2R3-MYB* genes in anthocyanin accumulation in peach fruits, consistent with previous studies (Petroni and Tonelli 2011, Xu et al. 2015). In summary, the effect of light on anthocyanin accumulation in fruit skin was mainly associated with activation of *PpMYB10.1*–*PpMYB10.3* and their downstream gene *PpGST*.

Peroxidase plays an important role in anthocyanin degradation in fruits. Thus, we investigated the impact of light on the expression of genes encoding peroxidase. Four homologs of the previously reported *BcProx01* involved in the degradation of anthocyanins (Zipor et al. 2005) have been identified in the peach genome (Figure S1A available as Supplementary data at *Tree Physiology* Online). Of these four peroxidase genes, two *PpePrx48* and *PpePrx22* were highly expressed in both sun-exposed and shaded peel tissues (Figure S1B available as Supplementary data at *Tree Physiology* Online), suggesting that light had no impact on the expression of the *PpePrx* genes.

### Transcription factor PpHYH was differentially expressed between the sun-exposed and shaded peel tissues

To get insight into the mechanism of light-induced accumulation of anthocyanin in the fruit exocarp, we conducted comparative transcriptome analysis to identify regulatory genes that were differentially expressed between the sun-exposed and shaded peel tissues of ‘MLWN’. A total of six libraries were sequenced and 22.0–27.0 million clean reads were generated for each library, with the Q30 value larger than 94% (Table S3 available as Supplementary data at *Tree Physiology* Online). Over 90% of clean reads were uniquely mapped to the peach reference genome. Pearson correlation coefficients between biological replicates of each sample were greater than 0.96 (Figure S2A available as Supplementary data at *Tree Physiology* Online). Principal component analysis showed that three biological replicates of each sample were clustered together (Figure S2B available as Supplementary data at *Tree Physiology* Online), suggesting a high consistency between biological replicates. In addition, we estimated the expression levels of anthocyanin structural and regulatory genes based on the RNA-seq data (Figure S3 available as Supplementary data at *Tree Physiology* Online), and the results were well consistent with those of qRT-PCR (Figure 1C and D). All these results indicated that the RNA-seq data generated in this study were suitable for conducting comparative transcriptome analysis.

A total of 629 DEGs, with 248 upregulated and 381 downregulated in the sun-exposed peel tissue, were identified (Figure S2C available as Supplementary data at *Tree Physiology* Online). Gene Ontology analysis showed that the DEGs were mainly involved in the biosynthesis and transport of secondary metabolites, signaling transduction and carbohydrate transport metabolism (Figure S2D available as Supplementary data at *Tree Physiology* Online). We screened that annotation of the DEGs based on the peach reference genome (Verde et al. 2013) and one encoding elongated hypocotyl 5-homolog (Prupe.1G208500), designated *PpHYH*, was identified. Notably, the light-response gene encoding elongated HY5 (Prupe.1G478400), which has been reported to regulate anthocyanin accumulation in the fruit exocarp in response to UVA and UVB irradiation (Zhao et al. 2021), was not included in the DEGs. Expression level of *PpHYH* in the sun-exposed side was approximately 3.0-fold higher than in the shaded side, while *PpHY5* showed a slightly lower level of expression in the sun-exposed side compared with the shade side (Figure S4 available as Supplementary data at *Tree Physiology* Online). Hence, the *PpHYH* gene was chosen for later functional analysis.

**Figure 2.**
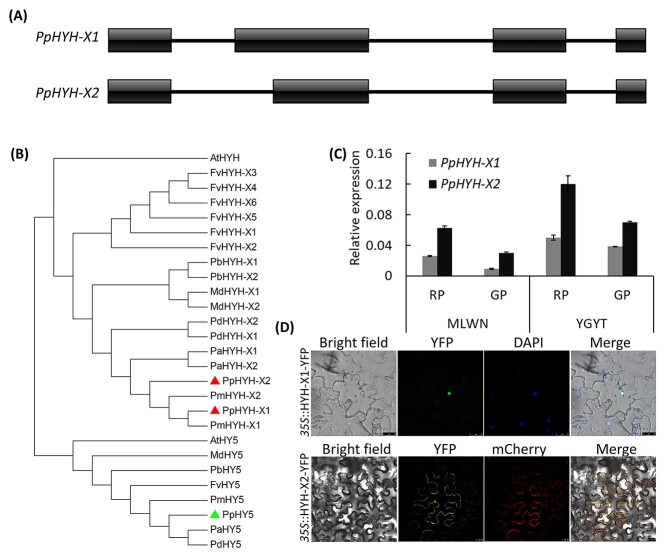
A light response gene *PpHYH* involved in anthocyanin accumulation in the exocap of peach fruits. (A) Genomic structure of *PpHYH*. The boxes and solid lines represent exons and introns, respectively. (B) Phylogenetic tree derived from amino acid sequences of *HYH* and *HY5* genes in peach and other plant species. The genes in peach are highlighted by solid triangles. All sequences are retrieved from NCBI database with accessions as follows: *P. persica* PpHY5 (XP_020411091.1), PpHYH-X1 (XP_020409867.1) and PpHYH-X2 (XP_007223521.1); *P. avium* PaHY5 (XP_021827650.1), PaHYH-X1 (XP_021812250.1) and PaHYH-X2 (XP_021812251.1); *Fragaria vesca* FvHY5 (XP_004291469.1), FvHYH-X1 (XP_011462860.1), FvHYH-X2 (XP_011462861.1), FvHYH-X3 (XP_011462862.1), FvHYH-X4 (XP_004297046.1), FvHYH-X5 (XP_011462863.1) and FvHYH-X6 (XP_011462864.1); *Malus domestica* MdHY5 (NP_001280752.1), MdHYH-X1 (XP_008369576.1) and MdHYH-X2 (XP_008369578.1); *Pyrus* × *bretschneideri* PbHY5 (QGP73826.1), PbHYH-X1 (XP_009353456.1) and PbHYH-X2 (XP_009353457.1); *P. mume* PmHY5 (XP_008219477.1), PmHYH-X1 (XP_008222315.1) and PmHYH-X2 (XP_008222316.1); *P. dulcis* PdHY5 (XP_034200964.1), PdHYH-X1 (XP_034200998.1) and PdHYH-X2 (XP_034200999.1). (C) Expression of *PpHYHs* in the sun-exposed and shaded peel tissues of ‘MLWN’ and ‘YGYT’. (D) Subcellular localization assay of PpHYHs in tobacco leaves.

The *PpHYH* gene had two distinct alternative splicing variants, designated *PpHYH-X1* and *PpHYH-X2* (Figure 2A). The second exon of *PpHYH-X1* is 84 bp longer than that of *PpHYH-X2* due to an alternative donor event. Alternative splicing of the *HYH* genes was also detected in other fruit crops such as plum, almond, sweet cherry, strawberry and apple (Figure 2B). Amino acid sequence alignment showed that all the *HYH* and *HY5* genes had a conserved bZIP domain (Figure S5 available as Supplementary data at *Tree Physiology* Online). The qRT-PCR analysis revealed that both *PpHYH-X1* and *PpHYH-X2* showed significantly higher levels of expression in the sun-exposed peel than in the shaded peel (Figure 2C), which is quite similar to the expression profiles of *PpMYB10.1–PpMYB10.3* mentioned above. Subcellular localization assay showed that PpHYH-X1 was located in the nucleus, while PpHYH-X2 was located in both the cytoplasmic membrane and the nucleus (Figure 2D). These results suggested that *PpHYH* could be involved in regulation of anthocyanin accumulation in the fruit exocarp via activating transcription of the *PpMYB10* genes.

### PpHYH interacts with PpBBX4 to induce transcription of anthocyanin-related structural and regulatory genes

**Figure 3.**
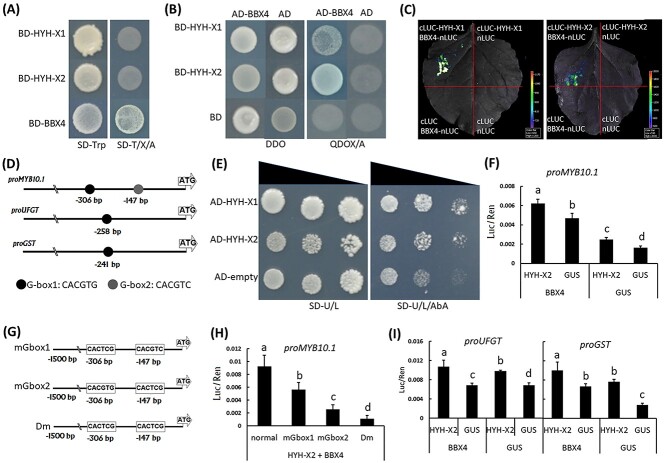
Transcriptional activity analysis of the *PpHYH* gene. (A) Auto-activation activity assay of PpHYH and PpBBX4 using Y2H. (B) Validation of the interaction between PpHYH and PpBBX4 using Y2H. (C) Validation of the interaction between PpHYH and PpBBX4 using firefly luciferase complementation assay. (D) Two G-box elements in the promoter sequences of *PpMYB10.1*. (E) Assessment of the binding ability of PpHYH to the promoter of *PpMYB10.1* using Y1H. (F) Assessment of the activation of PpHYH-X2 on the promoter of *PpMYB10.1* using the dual-luciferase reporter system. (G) Schematic diagram of three variants of the *PpMYB10.1* promoter that are generated by nucleotide mutation. (H) Assessment of the activation of PpHYH-X2 on various variants of the *PpMYB10.1* promoter using the dual-luciferase reporter system. (I) Assessment of the activation of PpHYH-X2 on the promoter of *PpUFGT* and *PpGST* using the dual-luciferase reporter system. The error bars in (F), (H) and (I) show SE of at least three biological replicates, and significant difference at *P* < 0.05 is indicted by different lowercase letters based on Fisher’s least significant difference (LSD) test.

Self-activation assay showed that PpHYH-X1 and PpHYH-X2 lacked of transcriptional activation activity in yeast (Figure 3A). Since HY5 requires co-factors to activate downstream genes (Stracke et al. 2010), we performed the Y2H library screening using the PpHYH-X1 as bait. As a result, one partner termed PpBBX4 (Prupe.4G156600) was identified. PpBBX4 was phylogenetically related to *Arabidopsis* AtLZF1 and apple MdCOL11 (Figure S6 available as Supplementary data at *Tree Physiology* Online) which are both involved in anthocyanin accumulation through interacting with HY5 (Bai et al. 2014, Plunkett et al. 2019). To validate whether PpBBX4 was the cofactor of PpHYH, self-activation assay was conducted and the results indicated that PpBBX4 had transcriptional activation activity in yeast (Figure 3A). Subsequently, the interaction between PpBBX4 and either PpHYH-X1 or PpHYH-X2 was validated using Y2H assay (Figure 3B) and firefly luciferase complementation experiment (Figure 3C). To identify the domains responsible for the HYH–BBX interaction, three PpHYH truncated fragments and two BBX4 truncated fragments were generated (Figure S7A available as Supplementary data at *Tree Physiology* Online). The PpHYH truncated fragments lacking the bZIP domain were not able to interact with PpBBX4 (Figure S7B available as Supplementary data at *Tree Physiology* Online). By contrast, the N-terminal of PpBBX4 containing the B-box domain strongly interacted with the C-terminal of PpHYH containing the bZIP domain. These results indicated that the B-box domain of PpBBX4 and the bZIP domain of PpHYH are responsible for their interaction.

Since *PpMYB10.1* has been reported to be crucial for anthocyanin accumulation in peach fruits (Rahim et al. 2014, Zhou et al. 2015, 2018), it was selected to test its interaction with the *PpHYH* gene. HY5 is known to bind to the G-box motif (CACGTG/C) (Shin et al. 2013). As shown in Figure 3D, two G-box motifs were detected in the promoter of *PpMYB10.1*. Y1H assay showed that both PpHYH-X1 and PpHYH-X2 were able to bind to the promoter of *PpMYB10.1* (Figure 3E). The dual-luciferase assay indicated that PpHYH-X2 co-infiltrated with PpBBX4 was able to activate transcription of *PpMYB10.1*, but PpHYH-X2 infiltrated alone could not (Figure 3F). Additionally, PpHYH-X1 co-infiltrated with PpBBX4 was also able to activate transcription of *PpMYB10.1*, but the transcriptional activation activity is lower than that of PpHYH-X2 co-infiltrated with PpBBX4 (Figure S8 available as Supplementary data at *Tree Physiology* Online). Thus, PpHYH-X2 was chosen for functional analysis later.

To validate whether PpHYH-X2 interacted with the G-box motif, we mutated two and three base pairs in the G-box1 and G-box2 motifs of *PpMYB10.1*, respectively, generating two mutant promoters, mGbox1 and mGbox2 (Figure 3G). Moreover, a third mutant promoter designated Dm was generated by combining the mutated base pairs in both mGbox1 and mGbox2. The dual-luciferase assay revealed a significant decrease in promoter activity for all these mutated promoters, and Dm had almost no promoter activity (Figure 3H). These results suggested that the PpHYH–PpBBX4 complex could activate transcription of *PpMYB10.1* via the binding of PpHYH to the G-box element.

**Figure 4.**
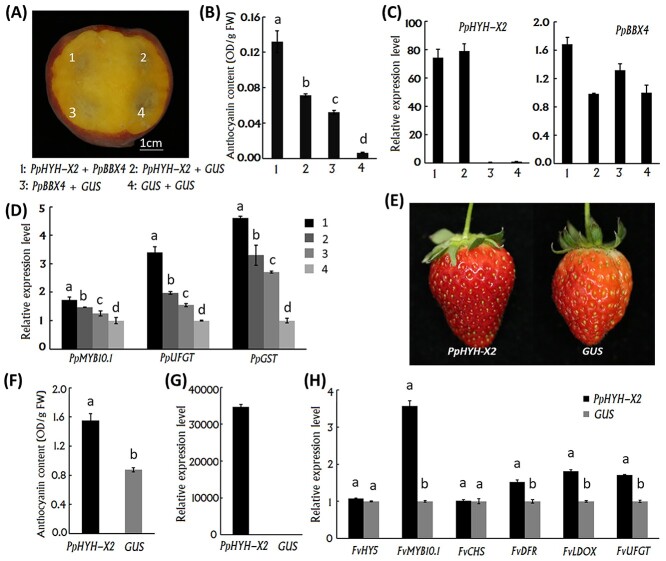
Functional analysis of the *PpHYH-X2* involved in anthocyanin accumulation in peach and strawberry fruits. (A) Peach fruits at the ripening stage infiltrated with *PpHYH-X2* or *PpBBX4* alone or both of them, with the *Gus* gene used as control. (B) Anthocyanin contents in peach flesh tissues around the same infiltration sites as indicated in (A). (C) Relative expression levels of *PpHYH-X2* and *PpBBX4* in peach flesh tissues around the infiltration sites that are indicated in (A). (D) Relative expression levels of *PpMYB10.1*, *PpUFGT* and *PpGST* in peach flesh tissues around the same infiltration sites as shown in (A). (E) Ectopic overexpression of *PpHYH-X2* in strawberry fruits during the post-veraison stage. (F) Anthocyanin contents in strawberry flesh tissues around the sites infiltrated with *PpHYH-X2.* (G) Relative expression levels of *PpHYH-X2* in strawberry flesh tissues around the sites infiltrated with *PpHYH-X2.* (H) Relative expression levels of anthocyanin-related genes in strawberry flesh tissues around the sites infiltrated with *PpHYH-X2.* The error bars in (B), (D), (F) and (H) show ±SE of three biological replicates, and significant difference at *P* < 0.05 is indicted by different lowercase letters based on LSD test.

Besides *PpMYB10.1*, anthocyanin biosynthesis structural genes such as *PpUFGT* and *PpGST* also contain the G-box element in the promoter region (Figure 3D). The dual-luciferase assay indicated that the PpHYH-PpBBX4 complex could activate transcription of *PpUFGT* and *PpGST* (Figure 3I). Taken together, the above results indicate that PpHYH interacts with PpBBX4 to induce the expression of anthocyanin-related structural and regulatory genes.

### PpHYH-X2 could induce anthocyanin accumulation in peach and strawberry fruits

The role of *PpHYH-X2* in anthocyanin coloration was tested by its transient overexpression in peach and strawberry fruits. Peach fruits of ‘XHJ’ at the ripening stage were transformed with *PpHYH-X2* or *PpBBX4* alone or with both of them. Transient overexpression of *PpHYH–X2* or *PpBBX4* alone or with both of them had no visual anthocyanin induction (Figure 4A), which might be attributed to the influence of yellow-fleshed background color. However, anthocyanin contents in the flesh tissues around the infiltration sites were significantly different among the four treatments, with the *PpBBX4/PpHYH-X2* treatment showing the highest concentration, followed by *PpHYH-X2/Gus, PpBBX4/Gus* and *Gus/Gus* (Figure 4B). Increased expression was observed for *PpHYH-X2* at infiltration sites 7 days after transformation with *PpHYH-X2* alone or both *PpBBX4* and *PpHYH-X2*, whereas the *PpBBX4* gene showed a slight difference in expression levels among all four treatments (Figure 4C). The expression patterns of *PpMYB10.1*, *PpUFGT* and *PpGST* in the flesh tissues around the infiltrated sites were in accordance with the anthocyanin contents (Figure 4D).

Moreover, the function of *PpHYH-X2* was validated by its ectopic overexpression in strawberry fruits during the post-veraison stage. After 7 days of infiltration, strawberry fruits infiltrated with *PpHYH-X2* appeared to be redder than the control infiltrated with *Gus* (Figure 4E). Anthocyanin content in the flesh tissues around the sites infiltrated with *PpHYH-X2* showed an approximately 1-fold increase compared with the control treatment of *Gus* (Figure 4F). The *PpHYH-X2* gene was highly expressed in the flesh tissues around the sites infiltrated with *PpHYH-X2*, while almost on expression was observed for the control treatment of *Gus* (Figure 4G). The expression of *FvMYB10*, an important regulator of anthocyanin synthesis (Chen et al. 2020), showed a 2.5-fold increase in the flesh tissues around the sites infiltrated with *PpHYH-X2*, while the expression level of *FvHY5* showed a slight increase (Figure 4H). In addition, the expression of anthocyanin biosynthetic pathway genes, such as *FvDFR*, *FvUFGT* and *FvUFGT*, were also significantly upregulated (Figure 4H), which is consistent with the increase in anthocyanin content. Taken together, all these results suggested that *PpHYH-X2* could induce anthocyanin accumulation via activating transcription of its downstream gene *MYB10*.

### PpHYH had auto-activation ability and its transcription was induced in response to light

**Figure 5.**
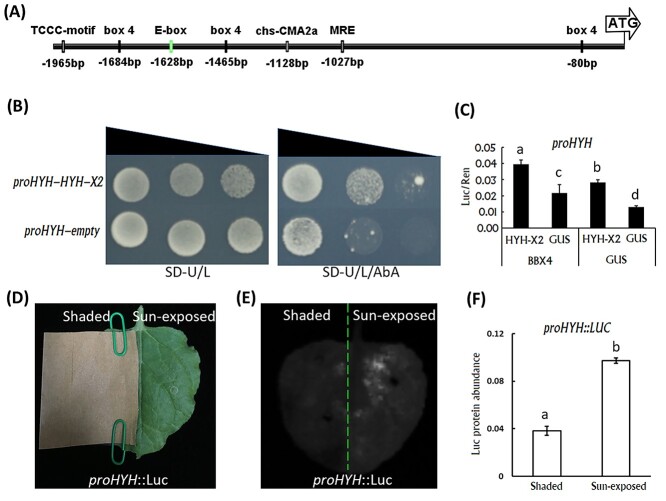
Identification and functional analysis of *cis*-elements in the promoter of *PpHYH*. (A) Schematic of *cis*-elements in the promoter sequences of *PpHYH*. E-box element is highlighted in green color, while the light response elements are indicated in black boxes. (B) Assay of the binding activity of PpHYH-X2 to its own promoter using Y1H. (C) Assessment of the activation of PpHYH-X2 on its own promoter using the dual-luciferase reporter system. (D) Assessment of the promoter activity of *PpHYH* via transient expression of the *Luc* gene fused with its promoter. The left side was covered with brown Kraft paper, while the right side was under normal light conditions. (E) The chemiluminescence image tobacco leaves shown in (D) that were taken 3 days after infiltration. (F) Quantification of the luciferase in the sun-exposed and shaded sides of the same tobacco leaf as shown in (D). The measurement was conducted 3 days after infiltration. The error bars in (C) and (F) show ±SE of at least three biological replicates, and significant difference at *P* < 0.05 is indicted by different lowercase letters based on LSD test.

The *HYH* and *HY5* genes are known to activate downstream genes via binding to G-box and E-box (CAATTG) *cis*-regulatory elements (Binkert et al. 2014). Since an E-box element 1630–1625 bp upstream of the start codon was identified in the promoter region of *PpHYH* (Figure 5A), we clarified whether PpHYH had auto-activation ability. Y1H assay showed that PpHYH-X2 could bind to its promoter (Figure 5B). Moreover, the dual-luciferase assay further confirmed that PpHYH-X2 was able to activate its own transcription and this auto-activation ability could be significantly enhanced by the presence of its co-factor PpBBX4 (Figure 5C).

Besides the E-box element, six light response elements were also identified in the promoter region of *PpHYH* (Figure 5A). To test whether the expression of *PpHYH* was induced by light, the promoter sequence 2-kb upstream of the start codon of *PpHYH* was fused to the luciferase reporter gene (*Luc*). The ProHYH::Luc construct was transiently transformed into both sides of tobacco leaf, and the left side was covered with brown kraft paper (Figure 5D). After 3 days of transformation, a strong chemiluminescence signal was detected in the light-exposed side, but with a very weak signal in the shaded side (Figure 5E). Quantification of luciferase protein abundance revealed a much higher level in the sun-exposed side than in the shaded side (Figure 5F). These results indicated that the expression of the *PpHYH* gene could be induced by light exposure.

### PpCOP1 had a negative impact on the auto-activation ability of PpHYH

**Figure 6.**
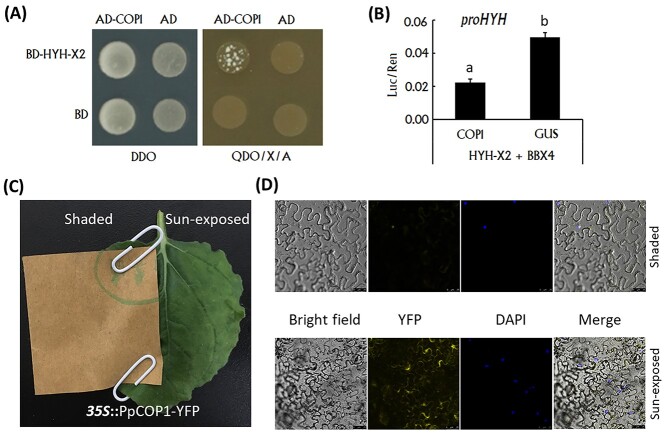
Functional analysis of the *PpCOP1* gene. (A) Validation of the interaction between PpCOP1 and PpHYH-X2 using Y2H. (B) Assay of the influence of PpCOP1 on the activation of the PpHYH-PpBBX4 complex using the dual-luciferase reporter system. (C) Transient expression of the *YFP* gene fused with the coding sequence of *PpCOP1* gene in tobacco leaves. The left side was covered with brown Kraft paper, while the right side was under normal light conditions. (D) Subcellular localization of PpCOP1. Expression of pFGC-eYFPPpCOP1 in bright field, YFP channel, DAPI channel, and merged channel, respectively. PpCOP1 is located in the nucleus under dark condition, while it transfers to cytosol under light condition. The error bars in (B) show ± SE of three biological replicates, and significant difference at *P* < 0.05 is indicted by different lowercase letters based on LSD test.

COP1 acts as a key repressor of light signaling in darkness through ubiquitination of various protein substrates to trigger their proteasomal degradation (Maier et al. 2013). The annotated peach genome (Verde et al. 2013) was screened and two *COP1* genes, designated *PpCOP1* (Prupe.5G031300) and *PpCOP2* (Prupe.4G013500), were identified. Like *PpHYH*, the *PpCOP2* gene had also two distinct alternative splicing variants (Figure S9A available as Supplementary data at *Tree Physiology* Online). Analysis of RNA-seq data revealed that *PpCOP1* showed a much higher level of expression in the sun-exposed peel tissues of ‘MLWN’ than did the *PpCOP2* gene (Figure S9B available as Supplementary data at *Tree Physiology* Online). Y2H assay indicated that PpHYH-X2 was able to interact with PpCOP1 (Figure 6A), but could not interact with both PpCOP2 variants (Figure S9C available as Supplementary data at *Tree Physiology* Online). The dual-luciferase assay showed that PpCOP1 was able to reduce the auto-activation ability of PpHYH-X2 (Figure 6B).

Light inhibits COP1 activity by triggering its export from the nucleus to the cytosol (Saijo et al. 2003). To uncover whether the activity of PpCOP1 is inhibited in the sun-exposed peel tissues, it was transiently transformed into both sides of tobacco leaf and the left side was covered with brown Kraft paper (Figure 6C). After 3 days of transformation, the PpCOP1 subcellular localization was examined. As a result, PpCOP1 was only localized in the nucleus under dark condition, but it appeared mainly in the cytosol under light condition (Figure 6D). These results suggested that PpCOP1 could be exported from the nucleus to the cytosol under light exposure, leading to the loss of its regulatory role in anthocyanin accumulation.

## Discussion

### Vacuolar transport is a rate-limiting step for anthocyanin accumulation in the exocarp of P. persica fruits

Light is one of the most important environmental factors that stimulate anthocyanin accumulation in plants (Takos et al. 2006, Jaakola 2013). In this study, almost no anthocyanins were detected in the shaded peel of both peach and nectarine fruits, while anthocyanin pigmentation appeared clearly in the sun-exposed peel. This indicates that sunlight is essential for anthocyanin accumulation in fruit peel of *P. persica*. Interestingly, the sun-exposed peel accumulated 2.8-fold higher levels of anthocyanins in nectarine cv. YGYT than in peach cv. MLWN. Peach fruits have fuzzy skin due to the presence of pubescence, while the pubescence is absent on the skin of nectarine fruits. Thus, fruit pubescence seems to have a negative impact on anthocyanin accumulation in fruit peel probably through reducing the intensity of light entering fruit surface area.

Although activation of anthocyanin biosynthesis pathway genes by the MBW complex is well known to be responsible for anthocyanin pigmentation in fruits (Allan and Espley 2018), no anthocyanin biosynthetic genes were consistently up-regulated in the sun-exposed peel of both peach and nectarine fruits. By contrast, an anthocyanin transporter gene *PpGST* was strongly activated in the sun-exposed peel of both peach and nectarine fruits. These results suggested that the transport of anthocyanins from the cytoplasm to the vacuole is a rate-limiting step for anthocyanin accumulation in the exocarp of peach fruits. This finding is consistent with previous studies that have demonstrated that loss-of-function mutation of the *PpGST* gene results in anthocyanin deficiency in flower and fruit peel of peach (Cheng et al. 2015, Zhao et al. 2020, Lu et al. 2021). Additionally, anthocyanins are synthesized in the cytosolic surface of the ER and they are likely broken down into phenolic acids and aldehyde by peroxidase at higher pH in vivo (Khunmuang et al. 2019). To prevent enzymatic degradation, anthocyanins are transported from the cytoplasm into the vacuole, an acidic environment, once synthesized in the ER. In this study, two peroxidase genes, *PpePrx48* and *PpePrx22*, were highly expressed in both sun-exposed and shaded peel tissues (Figure S1B available as Supplementary data at *Tree Physiology* Online), suggesting an occurrence of anthocyanin degradation in the cells of peach fruit peel. Therefore, anthocyanin accumulation depends on the balance between degradation and vacuolar transport in fruit peel of *P. persica*, which is in accordance with the previous finding that the *PpGST* gene plays an important role in anthocyanin pigmentation in flower and fruit peel of peach (Cheng et al. 2015, Zhao et al. 2020, Lu et al. 2021).

### Light induces anthocyanin accumulation by activating transcription of PpHYH rather than PpHY5 in fruit peel of P. persica

HY5 that is a key component of light signaling has been reported to play an important role in regulating anthocyanin accumulation in fruit crops such as apple and pear (Li et al. 2012, Bai et al. 2019, Li et al. 2020). A recent study shows that *PpHY5* (Prupe.1G478400) is a vital regulator of anthocyanin accumulation in peach fruits in response to UV lights (Zhao et al. 2021). However, our results show that the expression of *PpHY5* had no significant difference between the sun-exposed and shaded peel tissues of either peach or nectarine fruits. By contrast, the *PpHY5* homolog *PpHYH* showed an increased expression in the sun-exposed peel tissues of both peach and nectarine fruits. Notably, the *PpHYH* gene has two alternative splicing variants and such alternative splicing event is also present in *HYH* genes from a variety of species within Rosaceae. This suggests that alternative splicing of *HYH* genes is conserved and may have occurred in the ancestral Rosaceae progenitor. Alternative splicing of *HYH* genes has also been reported in *Physcomitrella patens*, *Arabidopsis*, *Glycine max* and *Zea mays*, but not in *Oryza sativa* (Li et al. 2017), suggesting that the AS event of *HYH* genes is species specific although it occurs in a variety of plant species. Like the *PpHY5* gene, the *PpCOP2* gene also has two alternative splicing variants, consistent with previous studies that reveal a high frequency of AS events of genes in response to environmental stimuli such as light stress (Wang et al. 2013, Wu et al. 2014).

The expression of the two alternative splicing variants of *PpHYH* is induced by light and both variants can activate transcription of *PpMYB10.1* by binding to the G-box element in the *PpMYB10.1* promoter. Thus, the two *PpHYH* variants seem to have similar role in the regulation of anthocyanin accumulation, which is consistent with a previous study that shows functional redundancy among all *HYH* variants involved in the regulation of hypocotyl development in *Arabidopsis* (Li et al. 2017). However, one thing worthy of noting is the difference in subcellular localization between the two *HYH* variants. As mentioned above, alternative splicing of *PpHYH* genes is conserved in the family Rosaceae. Thus, it is worth further study to address the function of the *PpHYH-X2* that is located in both the cytoplasmic membrane and the nucleus.

In this study, the activation of *PpHYH* on transcription of *PpMYB10.1* was validated using both Y1H and the dual-luciferase assays. Like *PpMYB10.1*, two other anthocyanin-activating *R2R3*-*MYB* genes, *PpMYB10.2* and *PpMYB10.3*, also showed higher levels of expression in the sun-exposed peel tissues than in the shaded peel tissues. Transient overexpression of *PpHYH* in peach fruits can activate transcription of *PpMYB10.1*, *PpMYB10.2* and *PpMYB10.3* (Figure S10A available as Supplementary data at *Tree Physiology* Online). Moreover, the dual-luciferase assay reveals that PpHYH is able to activate transcription of *PpMYB10.2* and *PpMYB10.3* (Figure S10B available as Supplementary data at *Tree Physiology* Online). All these results suggest that the *PpHYH* gene could activate transcription of all the three *PpMYB10* genes in vivo. Previous studies have indicated that these three *PpMYB10* genes are all able to induce anthocyanin accumulation (Rahim et al. 2014, Zhou et al. 2018). Hence, it is likely that *PpMYB10.1*, *PpMYB10.2* and *PpMYB10.3* are all involved in the regulation of anthocyanin accumulation in fruit peel of *P. perscia* under light condition. By contrast, only *PpMYB10.1* out of the three *PpMYB10* genes is involved in the regulation of anthocyanin accumulation in blood-fleshed peach fruit as either *PpMYB10.2* or *PpMYB10.3* is not expressed (Zhou et al. 2015). In blood-fleshed peach fruit, *PpHYH* shows no expression due to the lack of sunlight and transcription of *PpMYB10.1* is activated by the *NAC* gene *BL*. Moreover, our results show that PpHYH lacks self-activation but it is able to interact with the cofactor PpBBX4 to activate its target genes. This finding is consistent with a previous study that shows that *PyHY5* in response to light interacts with *PyBBX16* to activate transcription of *PyMYB10*, resulting in anthocyanin accumulation in pear fruit (Bai et al. 2019). It is worth noting that the bZIP domain of PpHYH has ability to interact with the B-box domain of PpBBX4, which is in contrast to a previous finding that shows an interaction between the bZIP domain of HYH and the C-terminal of PpBBX4 which lacks any bZIP domain (Lin et al. 2018).

*PpHYH* consists of several light-response elements in its promoter, thus its transcription is induced by light. A similar result has also been reported for *AtHY5* in *Arabidopsis* (Abbas et al. 2014). Moreover, *PpHYH* has self-activation activity by binding the E-box element in its promoter, similar to the mechanism underlying auto-activation of *MdHY5* in apple (An et al. 2017). These results infer functional conservation between the *HYH* and *HY5* genes in the regulation of anthocyanin accumulation. HY5 is a known target of COP1-meidated degradation (Deng et al. 1992). The peach genome consists of two copies of *COP1*, but only one PpCOP1 is able to interact with PpHYH. The dual-luciferase assay reveals that PpCOP1 attenuated the transcriptional activation of the PpHYH–PpBBX4 complex. These results suggest an important role of *PpCOP1* in the light signaling pathway in peach. The expression level of the *PpCOP1* gene in fruit peel is not affected by light. However, sunlight causes the nuclear efflux of PpCOP1 and its re-accumulation in the cytoplasm, similar to that reported for AtCOP1 (Vonarnim and Deng 1994). This is likely the main reason why anthocyanin pigmentation occurs in the sun-exposed peel but not in the shaded peel of *P. persica*.

**Figure 7.**
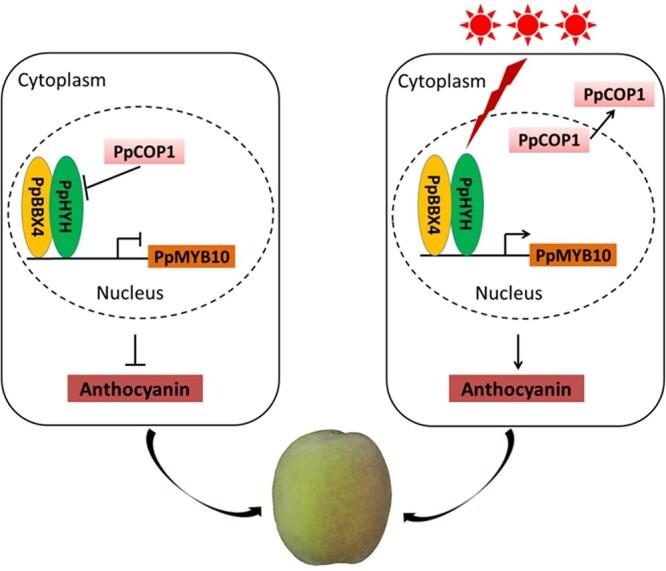
A proposed model for anthocyanin accumulation in the fruit exocarp of peach. In the shaded peel tissues (left), PpCOP1 accumulates in the nucleus to mediate the ubiquitin-dependent degradation of PpHYH. Thus transcription of *PpMYB10.1* cannot be activated, resulting in green coloration instead of anthocyanin coloration. In the sun-exposed peel tissues (right), sunlight inhibits PpCOP1 activity by triggering its export from the nucleus to the cytosol, and PpHYH along with its co-factor PpBBX4 activate transcription of *PpMYB10.1*, leading to anthocyanin red pigmentation. The red lightning symbol indicates that transcription of *PpHYH* is induced by sunlight.

Taking all the above results together, a model for light-induced anthocyanin pigmentation in fruit peel of *P. persica* is proposed (Figure 7). In the shaded peel tissues, PpHYH is degraded via a PpCOP1-dependent pathway, thus PpMYB10 genes cannot be activated, resulting in no anthocyanin accumulation. By contrast, light not only induces transcription of *PpHYH*, but also triggers the export of PpCOP1 from the nucleus to the cytoplasm. Thus, *PpHYH* along with its co-factor PpBBX4 activates transcription of *PpMYB10* genes to induce anthocyanin accumulation in the sun-exposed peel tissues. In *Arabidopsis*, the photoreceptors such as AtCRY1/2 and AtUVR8 interact with AtCOP1 to inhibit the ubiquitination degradation of HY5/HYH via the AtCOP1-dependent pathway (Yang et al. 2001, Zuo et al. 2011, Yin et al. 2015, Li et al. 2021). Besides HYH/HY5, anthocyanin-activating R2R3-MYBs AtPAP1/2 can also be ubiquitinated and subsequently degraded by AtCOP1 (Maier et al. 2013). In this study, photoreceptor genes, *PpCRY1/2* and *PpUVR8*, are also expressed in the sun-exposed and shaded peel tissues of *P. persica*. More studies are needed to clarify whether PpCRY1/2 and PpUVR8 are able to interact with PpCOP1 and participate in the degradation of PpMYB10 TFs in peach.

### The potential role of PpHYH in the regulation of flavonol accumulation in fruit peel of peach

Besides anthocyanins, flavonols also showed a higher level of accumulation in the sun-exposed peel tissues of *P. persica* (Figure S11 available as Supplementary data at *Tree Physiology* Online). In *Arabidopsis*, *AtMYB12* is proven to be a positive regulator of flavonol accumulation by directly activating transcription of *AtFLS* (Mehrtens et al. 2005). AtHY5 is able to activate transcription of *AtFLS* and *AtMYB12* by binding to the ACE element in their promoters (Shin et al. 2007, Stracke et al. 2010). In this study, we found that *PpMYB12* (Prupe.8G270000) and *PpFLS* (Prupe.1G502800) are orthologs of *AtMYB12* and *AtFLS*, respectively (Figure S12A and B available as Supplementary data at *Tree Physiology* Online). The expression levels of *PpMYB12* and *PpFLS* were higher in the sun-exposed peel tissues than in the shaded peel tissues (Figure S12C available as Supplementary data at *Tree Physiology* Online). The *PpFLS* promoter contains three G-box elements in the upstream regions of −1243 to −1238 bp, −341 to −336 bp and −261 to −256 bp, respectively. The *PpMYB12* promoter lacks of any G-box element, but contains an E-box element (−1596 to −1591 bp). Therefore, it is worthy of further study to clarify whether PpHYH could activate transcription of flavonol-related regulatory and structural genes to induce flavonol accumulation in fruit peel of *P. persica* under light condition.

## Authors’ contributions

Y.H. and L.Z. planned and designed the experiments. L.Z., J.S. and Y.C. performed the experiments. F.W., Y.Z. and H.H. collected fruit samples. Q.Y., Y.Z. and J.L. performed the sequence analysis. L.Z. wrote the paper. C.O.O. and Y.H. revised the manuscript.

## Conflict of interest

The authors declare no competing interests.

## Data availability statement

All data can be found online in the main text and supporting information materials.

## Supplementary Material

Supplementary_file_tpac025

## References

[ref1] Abbas N , Maurya JP, Senapati D, Gangappa SN, Chattopadhyay S (2014) *Arabidopsis* CAM7 and HY5 physically interact and directly bind to the HY5 promoter to regulate its expression and thereby promote photomorphogenesis. Plant Cell 26:1036–1052. 24610722 10.1105/tpc.113.122515 PMC4001367

[ref2] Allan AC , Espley RV (2018) MYBs drive novel consumer traits in fruits and vegetables. Trends Plant Sci 23:693–705. 30033210 10.1016/j.tplants.2018.06.001

[ref3] Amoussa AMO , Sanni A, Lagnika L (2015) Antioxidant activity and total phenolic, flavonoid and flavonol contents of the bark extracts of *Acacia ataxacantha*. J Pharmacogn Phytochem 4:172–178.

[ref4] An JP , Qu FJ, Yao JF, Wang XN, You CX, Wang XF, Hao YJ (2017) The bZIP transcription factor MdHY5 regulates anthocyanin accumulation and nitrate assimilation in apple. Hortic Res 4:1–9. 10.1038/hortres.2017.23 PMC5461414 28611922

[ref5] Bai S , Saito T, Honda C, Hatsuyama Y, Ito A, Moriguchi T (2014) An apple B-box protein, MdCOL11, is involved in UV-B- and temperature-induced anthocyanin biosynthesis. Planta 240:1051–1062. 25074586 10.1007/s00425-014-2129-8

[ref6] Bai S , Tao R, Tang Y et al. (2019) BBX16, a B-box protein, positively regulates light-induced anthocyanin accumulation by activating *MYB10* in red pear. Plant Biotechnol J 17:1985–1997. 30963689 10.1111/pbi.13114 PMC6737026

[ref7] Binkert M , Kozma BL, Terecskei K, De Veylder L, Nagy F, Ulm R (2014) UV-B-responsive association of the *Arabidopsis* bZIP transcription factor ELONGATED HYPOCOTYL5 with target genes, including its own promoter. Plant Cell 26:4200–4213. 25351492 10.1105/tpc.114.130716 PMC4247584

[ref8] Bursch K , Toledo OG, Pireyre M, Lohr M, Braatz C, Johansson H (2020) Identification of BBX proteins as rate-limiting cofactors of HY5. Nat Plants 6:921. 32661279 10.1038/s41477-020-0725-0

[ref9] Cabrita L , Petrov V, Pina F (2014) On the thermal degradation of anthocyanidins: Cyanidin. RSC Adv 4:18939–18944.

[ref10] Chen G , Xu P, Pan J, Li Y, Zhou J, Kuang H, Lian H (2020) Inhibition of FvMYB10 transcriptional activity promotes color loss in strawberry fruit. Plant Sci 298:110578. 32771176 10.1016/j.plantsci.2020.110578

[ref11] Cheng J , Liao L, Zhou H, Gu C, Wang L, Han Y (2015) A small indel mutation in an anthocyanin transporter causes variegated colouration of peach flowers. J Exp Bot 66:7227–7239. 26357885 10.1093/jxb/erv419 PMC4765791

[ref12] Deng XW , Matsui M, Wei N, Wagner D, Chu AM, Feldmann KA, Quail PH (1992) COP1, an Arabidopsis regulatory gene, encodes a protein with both a zinc-binding motif and a Gβ homologous domain. Cell 71:791–801. 1423630 10.1016/0092-8674(92)90555-q

[ref13] Fuleki T , Francis FJ (1968) Quantitative methods for anthocyanins. 2. Determination of total anthocyanin and degradation index for cranberry juice. J Food Sci 33:78–83.

[ref14] He J , Giusti MM (2010) Anthocyanins: natural colorants with health-promoting properties. Annu Rev Food Sci Technol 1:163–187. 22129334 10.1146/annurev.food.080708.100754

[ref15] Holton TA , Cornish EC (1995) Genetics and biochemistry of anthocyanin biosynthesis. Plant Cell 7:1071–1083. 12242398 10.1105/tpc.7.7.1071 PMC160913

[ref16] Jaakola L (2013) New insights into the regulation of anthocyanin biosynthesis in fruits. Trends Plant Sci 18:477–483. 23870661 10.1016/j.tplants.2013.06.003

[ref17] Jiao Y , Lau OS, Deng XW (2007) Light-regulated transcriptional networks in higher plants. Nat Rev Genet 8:217–230. 17304247 10.1038/nrg2049

[ref18] Khunmuang S , Kanlayanarat S, Wongs AC, Meir S, Philosoph HS, Oren SM, Ovadia R, Buanong M (2019) Ethylene induces a rapid degradation of petal anthocyanins in cut vanda 'Sansai Blue' orchid flowers. Front Plant Sci 10:1004. 31447870 10.3389/fpls.2019.01004 PMC6696881

[ref19] Langmead B , Salzberg S (2012) Fast gapped-read alignment with bowtie 2. Nat Methods 9:357–359. 22388286 10.1038/nmeth.1923 PMC3322381

[ref20] Lau OS , Deng XW (2012) The photomorphogenic repressors COP1 and DET1: 20 years later. Trends Plant Sci 17:584–593. 22705257 10.1016/j.tplants.2012.05.004

[ref21] Li C , Zheng L, Zhang J, Lv Y, Liu J, Wang X, Palfalvi G, Wang G, Zhang Y (2017) Characterization and functional analysis of four HYH splicing variants in *Arabidopsis* hypocotyl elongation. Gene 619:44–49. 28389360 10.1016/j.gene.2017.04.001

[ref23] Li Y , Xu P, Chen G, Wu J, Liu Z, Lian H (2020) FvbHLH9 functions as a positive regulator of anthocyanin biosynthesis by forming a HY5–bHLH9 transcription complex in strawberry fruits. Plant Cell Physiol 61:826–837. 32016380 10.1093/pcp/pcaa010

[ref22] Li Y , Shi Y, Li M, Fu D, Wu S, Li J, Gong Z, Liu H, Yang S (2021) The CRY2–COP1–HY5–BBX7/8 module regulates blue light-dependent cold acclimation in Arabidopsis. Plant Cell 33:3555–3573. 34427646 10.1093/plcell/koab215 PMC8566302

[ref24] Li YY , Mao K, Zhao C, Zhao XY, Zhang HL, Shu HR, Hao YJ (2012) MdCOP1 ubiquitin E3 ligases interact with MdMYB1 to regulate light-induced anthocyanin biosynthesis and red fruit coloration in apple. Plant Physiol 160:1011–1022. 22855936 10.1104/pp.112.199703 PMC3461526

[ref25] Lightbourn GJ , Griesbach RJ, Novotny JA, Clevidence BA, Rao DD, Stommel JR (2008) Effects of anthocyanin and carotenoid combinations on foliage and immature fruit color of *Capsicum annuum* L. J Hered 99:105–111. 18222931 10.1093/jhered/esm108

[ref26] Lin F , Jiang Y, Li J, Yan T, Fan L, Liang J, Chen ZJ, Xu D, Deng XW (2018) B-Box Domain Protein28 negatively regulates photomorphogenesis by repressing the activity of transcription factor HY5 and undergoes COP1-mediated degradation. Plant Cell 30:2006–2019. 30099385 10.1105/tpc.18.00226 PMC6181009

[ref27] Love MI , Huber W, Anders S (2014) Moderated estimation of fold change and dispersion for RNA-seq data with DESeq2. Genome Biol 15:1–21. 10.1186/s13059-014-0550-8 PMC4302049 25516281

[ref28] Lu Z , Cao H, Pan L, Niu L, Wei B, Cui G, Wang L, Yao JL, Zeng W, Wang Z (2021) Two loss-of-function alleles of the *glutathione S-transferase* (*GST*) gene cause anthocyanin deficiency in flower and fruit skin of peach (*Prunus persica*). Plant J 107:1320–1331. 33964100 10.1111/tpj.15312

[ref29] Maier A , Schrader A, Kokkelink L et al. (2013) Light and the E3 ubiquitin ligase COP 1/SPA control the protein stability of the MYB transcription factors PAP1 and PAP2 involved in anthocyanin accumulation in Arabidopsis. Plant J 74:638–651. 23425305 10.1111/tpj.12153

[ref30] Marinova K , Pourcel L, Weder B, Schwarz M, Barron D, Routaboul JM, Debeaujon I, Klein M (2007) The *Arabidopsis* MATE transporter TT12 acts as a vacuolar flavonoid/H+-antiporter active in proanthocyanidin-accumulating cells of the seed coat. Plant Cell 19:2023–2038. 17601828 10.1105/tpc.106.046029 PMC1955721

[ref31] Mehrtens F , Kranz H, Bednarek P, Weisshaar B (2005) The Arabidopsis transcription factor MYB12 is a flavonol-specific regulator of phenylpropanoid biosynthesis. Plant Physiol 138:1083–1096. 15923334 10.1104/pp.104.058032 PMC1150422

[ref32] Osterlund MT , Hardtke CS, Wei N, Deng XW (2000) Targeted destabilization of HY5 during light-regulated development of Arabidopsis. Nature 405:462–466. 10839542 10.1038/35013076

[ref33] Petroni K , Tonelli C (2011) Recent advances on the regulation of anthocyanin synthesis in reproductive organs. Plant Sci 181:219–229. 21763532 10.1016/j.plantsci.2011.05.009

[ref34] Plunkett BJ , Henry-Kirk R, Friend A et al. (2019) Apple B-box factors regulate light-responsive anthocyanin biosynthesis genes. Sci Rep 9:17762. 31780719 10.1038/s41598-019-54166-2 PMC6882830

[ref35] Rahim MA , Busatto N, Trainotti L (2014) Regulation of anthocyanin biosynthesis in peach fruits. Planta 240:913–929. 24827911 10.1007/s00425-014-2078-2

[ref36] Saijo Y , Sullivan JA, Wang HY, Yang JP, Shen YP, Rubio V, Ma LG, Hoecker U, Deng XW (2003) The COP1–SPA1 interaction defines a critical step in phytochrome A-mediated regulation of HY5 activity. Genes Dev 17:2642–2647. 14597662 10.1101/gad.1122903 PMC280614

[ref37] Sass-Kiss A , Kiss J, Milotay P, Kerek MM, Toth MM (2005) Differences in anthocyanin and carotenoid content of fruits and vegetables. Food Res Int 38:1023–1029.

[ref38] Shin DH , Choi M, Kim K, Bang G, Cho M, Choi SB, Choi G, Park YI (2013) HY5 regulates anthocyanin biosynthesis by inducing the transcriptional activation of the MYB75/PAP1 transcription factor in Arabidopsis. FEBS Lett 587:1543–1547. 23583450 10.1016/j.febslet.2013.03.037

[ref39] Shin J , Park E, Choi G (2007) PIF3 regulates anthocyanin biosynthesis in an HY5-dependent manner with both factors directly binding anthocyanin biosynthetic gene promoters in Arabidopsis. Plant J 49:981–994. 17319847 10.1111/j.1365-313X.2006.03021.x

[ref40] Shitan N , Yazaki K (2013) New insights into the transport mechanisms in plant vacuoles. Int Rev Cell Mol Biol 305:383–433. 23890387 10.1016/B978-0-12-407695-2.00009-3

[ref41] Stracke R , Favory JJ, Gruber H, Bartelniewoehner L, Bartels S, Binkert M, Funk M, Weisshaar B, Ulm R (2010) The *Arabidopsis* bZIP transcription factor HY5 regulates expression of the *PFG1/MYB12* gene in response to light and ultraviolet-B radiation. Plant Cell Environ 33:88–103. 19895401 10.1111/j.1365-3040.2009.02061.x

[ref42] Sun Y , Li H, Huang JR (2012) Arabidopsis TT19 functions as a carrier to transport anthocyanin from the cytosol to tonoplasts. Mol Plant 5:387–400. 22201047 10.1093/mp/ssr110

[ref43] Takos AM , Jaffe FW, Jacob SR, Bogs J, Robinson SP, Walker AR (2006) Light-induced expression of a *MYB* gene regulates anthocyanin biosynthesis in red apples. Plant Physiol 142:1216–1232. 17012405 10.1104/pp.106.088104 PMC1630764

[ref44] Tong Z , Gao Z, Wang F, Zhou J, Zhang Z (2009) Selection of reliable reference genes for gene expression studies in peach using real-time PCR. BMC Mol Biol 10:1–13. 19619301 10.1186/1471-2199-10-71 PMC3224724

[ref45] Verde I , Abbott AG, Scalabrin S et al. (2013) The high-quality draft genome of peach (*Prunus persica*) identifies unique patterns of genetic diversity, domestication and genome evolution. Nat Genet 45:487–494. 23525075 10.1038/ng.2586

[ref46] Vonarnim AG , Deng XW (1994) Light inactivation of Arabidopsis photomorphogenic repressor COP1 involves a cell-specific regulation of its nucleocytoplasmic partitioning. Cell 79:1035–1045. 8001131 10.1016/0092-8674(94)90034-5

[ref47] Wang L , Zhao S, Gu C, Zhou Y, Zhou H, Ma J, Cheng J, Han Y (2013) Deep RNA-Seq uncovers the peach transcriptome landscape. Plant Mol Biol 83:365–377. 23783411 10.1007/s11103-013-0093-5

[ref48] Weldon WFR (1901) Mendel's laws of alternative inheritance in peas. Biometrika 1:228–254.

[ref49] Winkel SB (2002) Biosynthesis of flavonoids and effects of stress. Curr Opin Plant Biol 5:218–223. 11960739 10.1016/s1369-5266(02)00256-x

[ref50] Wu HP , Su YS, Chen HC, Chen YR, Wu CC, Lin WD, Tu SL (2014) Genome-wide analysis of light-regulated alternative splicing mediated by photoreceptors in *Physcomitrella patens*. Genome Biol 15:1–18. 10.1186/gb-2014-15-1-r10 PMC4054894 24398233

[ref51] Xu W , Dubos C, Lepiniec L (2015) Transcriptional control of flavonoid biosynthesis by MYB–bHLH–WDR complexes. Trends Plant Sci 20:176–185. 25577424 10.1016/j.tplants.2014.12.001

[ref52] Yang HQ , Tang RH, Cashmore AR (2001) The signaling mechanism of Arabidopsis CRY1 involves direct interaction with COP1. Plant Cell 13:2573–2587. 11752373 10.1105/tpc.010367 PMC139474

[ref53] Yin R , Arongaus AB, Binkert M, Ulm R (2015) Two distinct domains of the UVR8 photoreceptor interact with COP1 to initiate UV-B signaling in Arabidopsis. Plant Cell 27:202–213. 25627067 10.1105/tpc.114.133868 PMC4330580

[ref54] Zhang Y , Zheng S, Liu Z, Wang L, Bi Y (2011) Both HY5 and HYH are necessary regulators for low temperature-induced anthocyanin accumulation in Arabidopsis seedlings. J Plant Physiol 168:367–374. 20932601 10.1016/j.jplph.2010.07.025

[ref55] Zhao Y , Dong W, Zhu Y, Allan AC, Kui LW, Xu C (2020) *PpGST1*, an anthocyanin-related glutathione S-transferase gene, is essential for fruit coloration in peach. Plant Biotechnol J 18:1284–1295. 31693790 10.1111/pbi.13291 PMC7152611

[ref56] Zhao Y , Min T, Chen M, Wang H, Zhu C, Jin R, Allan AC, Lin WK, Xu C (2021) The photomorphogenic transcription factor PpHY5 regulates anthocyanin accumulation in response to UVA and UVB irradiation. Front Plant Sci 11:2295. 10.3389/fpls.2020.603178 PMC7847898 33537042

[ref57] Zhou H , Kui LW, Wang H, Gu C, Dare AP, Espley RV, He H, Allan AC, Han Y (2015) Molecular genetics of blood-fleshed peach reveals activation of anthocyanin biosynthesis by NAC transcription factors. Plant J 82:105–121. 25688923 10.1111/tpj.12792

[ref58] Zhou H , Liao L, Xu S, Ren F, Zhao J, Ogutu C, Wang L, Jiang Q, Han Y (2018) Two amino acid changes in the R3 repeat cause functional divergence of two clustered MYB10 genes in peach. Plant Mol Biol 98:169–183. 30155830 10.1007/s11103-018-0773-2

[ref59] Zhou Y , Zhou H, Lin WK, Vimolmangkang S, Espley RV, Wang L, Allan AC, Han Y (2014) Transcriptome analysis and transient transformation suggest an ancient duplicated MYB transcription factor as a candidate gene for leaf red coloration in peach. BMC Plant Biol 14:1–13. 25551393 10.1186/s12870-014-0388-y PMC4302523

[ref60] Zipor G , Duarte P, Carqueijeiro I et al. (2005) In planta anthocyanin degradation by a vacuolar class III peroxidase in *Brunfelsia calycina * flowers. New Phytol 205:653–665. 10.1111/nph.13038 25256351

[ref61] Zuo Z , Liu H, Liu B, Liu X, Lin C (2011) Blue light-dependent interaction of CRY2 with SPA1 regulates COP1 activity and floral initiation in Arabidopsis. Curr Biol 21:841–847. 21514160 10.1016/j.cub.2011.03.048 PMC3150455

