# Supplementary material for: PpHYH is responsible for light-induced anthocyanin accumulation in fruit peel of Prunus persica
Source: Tree Physiol. 2022 Feb 26;42(8):1662–77. doi: 10.1093/treephys/tpac025 (PMC9366866; doi:10.1093/treephys/tpac025)
Supplement: Supplementary_file_tpac025 [file supplementary_file_tpac025.docx]

Table S1 Primers used for qRT-PCR analyses in this study

| **Name** | **Gene ID** | **Forward (5’→3’)** | **Reverse (5’→3’)** |
| --- | --- | --- | --- |
| PpCHS | Prupe.1G002900 | AACAAGGGTGCTCGTGTTCTC | GCTGCACCATCACCGAATAAG |
| PpCHI | Prupe.2G225200 | GAGATCGTTACAGGTCCATTTG | GTGGGAAGTTTTGATCCTTGA |
| PpF3H | Prupe.7G168300 | GGACTGGACACAGAGGCATT | AATTGTGCCTGGGTCAGTGT |
| PpF3’H | Prupe.5G203600 | CTCTCGCTCAAAGAGGATGC | CCATTCCACTGTGCTTGATG |
| PpDFR | Prupe.1G376400 | CGCCTCCAAGACTCTAGCTG | CCAGTGAGTGGGGAAAGTCC |
| PpLDOX | Prupe.5G086700 | AGGAGTTGAAGAAGGCAGCA | GCCTGGTCATTGGCATACTT |
| PpUFGT | Prupe.2G324700 | GTCCTAGCCCATGCTTCAGTC | GATCTCCAACACGTCCTCCAC |
| PpGST | Prupe.3G013600 | ACTTCAACTTCTGGTGCTGC | GCTGCTCTTTGATAATCTTTCC |
| PpMYB10.1 | Prupe.3G163100 | GAAATGATTGGTGGGAAACC | GTCCTTCTTCTGAAACATTGGT |
| PpMYB10.2 | Prupe.3G163000 | CACCATCAACAAGGATTGGA | CCGATTGTGGCATATCATCA |
| PpMYB10.3 | Prupe.3G163300 | AAGGCCACAACCAAGAAGA | AACCCAAGACCAGAACCTGT |
| PpbHLH3 | Prupe.8G242100 | TCTTGTTCAGCGTTCCGTTCCT | TTGGCGCTGAGCTCATCTTGTG |
| PpbHLH33 | Prupe.2G170100 | GGCCTGTGACATTGACGAAACTG | CTATAAGGGCATCTCATCTCTA |
| PpActin | Prupe.8G132000 | TGCCATTGAAATCCTGAAAC | ACCAATTGGATCATCCTCCT |
| PpHYH-X1 | Prupe.1G208500 | AAGAGCAAACTAGACGTTCCTTGGT | TTCATCGCTCTCTTCTGGAACATT |
| PpHYH-X2 | Prupe.1G208500 | aaacaacaagaagagcaaactagaa | CGTTTTGCAGGAAAGCCAGAC |
| FvHY5 | gene11666 | GAAGATTCAATGCCAACAAC | CATCATCATCATCACCATCA |
| FvMYB10 | gene31413 | TCAAATCAGGCTTAAACAGA | TTAAAGACCACCTGTTTCCT |
| FvCHS | gene26825 | GGCTCACCGTCGAGACCG | GGTGAACCCAGATACCTTC |
| FvDFR | gene15174 | CGATTCACGACATTGCGAAATT | CTCAAACCCCATCTCTTTCAGCTT |
| FvLDOX | gene32347 | GAAGTGCGTACCCAACTCCATCGT | ACCTTCTCCTTGTTGACGAGCCC |
| FvUFGT | gene12591 | CTAAGCAAAGGAAAGTTGAACGGAAT | TCCAACCGCAATGTGTTACAAA |
| Fv26S | gene11892 | TAACCGCATCAGGTCTCCAA | CTCGAGCAGTTCTCCGACAG |

Table S2 Primers used for vector construction in this study

| **Primer name** | **Direction (5’→3’)** | **Enzyme site** |
| --- | --- | --- |
| YFP-HYH-F | TTACATTTACAATTAC ATGTCAGTCCCCATCAAACCAG | BamHI |
| YFP-HYH-R | CCTCGCCCTTGCCCAT CTTAACTGATCCTGCCTTTGGC | BamHI |
| YFP-COP1-F | ACAATTACATTTACAATTAC atggaggagtgctcgactggg | BamHI |
| YFP-COP1-R | AGCTCCTCGCCCTTGCCCAT agcagcaagaaccagcactt | BamHI |
| BD-HYH-F | CATATGGCCATGGAGGCC ATGTCAGTCCCCATCAAACCAGG | EcoRI |
| BD-HYH-R | CGGCCGCTGCAGGTCGACG TTACTTAACTGATCCTGCCTTTGGC | BamHI |
| BD-HYH-CF | GCATATGGCCATGGAGGCC gtgtctgctcaacaagctcg | EcoRI |
| BD-HYH-NR | CGGCCGCTGCAGGTCGACG tta cctgttccttagcaatc | BamHI |
| BD-BBX4-F | GCATATGGCCATGGAGGCC atgaagatacagtgcaacgt | EcoRI |
| BD-BBX4-R | CGGCCGCTGCAGGTCGACG ctagaattgcctacgacgtt | BamHI |
| BD-BBX4-NR | CATATGGCCATGGAGGCC CTA GAGCAAAAACCTCCGGT | BamHI |
| BD-COP1-F | GCATATGGCCATGGAGGCC atggaggagtgctcgactgg | EcoRI |
| BD-COP1-R | CGGCCGCTGCAGGTCGACG ttaagcagcaagaaccagca | BamHI |
| AD-HYH-F | Gccatggaggccagtgaattc ATGTCAGTCCCCATCAAACCAG | EcoRI |
| AD-HYH-R | Cagctcgagctcgatggatcc TTACTTAACTGATCCTGCCTTTG | BamHI |
| AD-HYH-CF | Gccatggaggccagtgaattc gtgtctgctcaacaagctcg | EcoRI |
| AD-HYH-NR | Cagctcgagctcgatggatcc tta cctgttccttagcaatc | BamHI |
| AD-BBX4-F | Gccatggaggccagtgaattc atgaagatacagtgcaacgt | EcoRI |
| AD-BBX4-R | Cagctcgagctcgatggatcc ctagaattgcctacgacgtt | BamHI |
| pAbAi-MYB10.1-F | AAGCTTGAATTCGAGCTC GATATCTGAAGACAGTTAAGCCGCG | KpnI |
| pAbAi-MYB10.1-R | TACATACAGAGCACATGC CTTCTTGTTGGCCAGCGTTGTAT | XhoI |
| pAbAi-HYH-F | AAGCTTGAATTCGAGCTC GGATGTACAGCATGCAAGCAC | KpnI |
| pAbAi-HYH-R | TACATACAGAGCACATGC GGCTTTAGCAGCTAGCTAGCTCT | XhoI |
| cLUC-HYH-F | cccggggcggtacccgggat atgtcagtccccatcaaacca | BamHI |
| cLUC-HYH-R | gtagtccatttgttggatcc ttacttaactgatcctgcct | BamHI |
| nLUC-BBX4-F | acgagctcggtacccgggat atgaagatacagtgcaacgt | BamHI |
| nLUC-BBX4-R | gcgtacgagatctggtcgac gaattgcctacgacgttTTG | SalI |
| pSAK277-HYH-F | gacactagtggatccaaaATGTCAGTCCCCATCAAACCAGG | EcoRI |
| pSAK277-HYH-R | gactctagaagtactTTACTTAACTGATCCTGCCTTTGGCT | XhoI |
| pSAK277-BBX4-F | ctagtggatccaaagaattc atgaagatacagtgcaacgt | EcoRI |
| pSAK277-BBX4-R | tcgagaagctttttgaattc ctagaattgcctacgacgtt | EcoRI |
| pSAK277-MYB10.1-F | ctagtggatccaaagaattc ATGGAGGGCTATAACTTGGG | EcoRI |
| pSAK277-MYB10.1-R | tcgagaagctttttgaattc TTAATGATTCCAAAAGTCCA | EcoRI |
| pSAK277-bHLH3-F | ctagtggatccaaagaattc ATGGCTGCACCGCCAAGT | EcoRI |
| pSAK277-bHLH3-R | tcgagaagctttttgaattc CTAGGAATCAGATTGGGGAATT | EcoRI |
| pGreen-MYB10.1-F | ctatagggcgaattgggtacc GATATCTGAAGACAGTTAAGCC | KpnI |
| pGreen-MYB10.1-R | Atcgataccgtcgacctcgag CTTCTTGTTGGCCAGCGTTGTAT | XhoI |
| pMYB10.1-MGBox-F | CAcacgtcCTGACACTAACAAACTCTTAGCTAACTGTT | NA |
| pMYB10.1-MGBox-R | TTAGTGTCAGgacgtgTGGTTCACGTATCACAGCTGTAATT | NA |
| pMYB10.1-MGbox2-F | AGACATTGcactcgACTGGCCTTGTAGCCTTAATCTG | NA |
| pMYB10.1-MGbox2-R | CAGTcgagtgCAATGTCTCGTGTCCACAGAAGTG | NA |
| pGreen-HYH-F | ctatagggcgaattgggtacc GGATGTACAGCATGCAAGCAC | KpnI |
| pGreen-HYH-R | Atcgataccgtcgacctcgag GGCTTTAGCAGCTAGCTAGCTCT | XhoI |
| pGreen-UFGT-F | ctatagggcgaattgggtacc ggcagctacaattgtcaaac | KpnI |
| pGreen-UFGT-R | atcgataccgtcgacctcgag atatgtatgagctaataaga | XhoI |
| pGreen-GST-F | ctatagggcgaattgggtacc atggactgcgcattttctct | KpnI |
| pGreen-GST-R | atcgataccgtcgacctcgag cttgttatattaatatctct | XhoI |

Table S3 Summary of RNA-seq data quality

| Sample* | No. of total reads | No. of clean reads | Mapped reads** | Uniq-mapped reads** | Multiple-mapped reads** | GC content | %≥Q30 |
| --- | --- | --- | --- | --- | --- | --- | --- |
| GP1 | 54,733,494 | 27,366,747 | 50,857,777 (92.92%) | 49,425,989 (90.30%) | 1,431,788 (2.62%) | 45.95% | 94.01% |
| GP2 | 51,447,012 | 25,723,506 | 48,160,585 (93.61%) | 46,822,993 (91.01%) | 1,337,592 (2.60%) | 45.70% | 94.47% |
| GP3 | 45,390,036 | 22,695,018 | 42,662,542 (93.99%) | 41,466,801 (91.36%) | 1,195,741 (2.63%) | 45.79% | 94.48% |
| RP1 | 50,332,708 | 25,166,354 | 46,656,632 (92.70%) | 45,313,396 (90.03%) | 1,343,236 (2.67%) | 45.49% | 94.38% |
| RP2 | 45,705,342 | 22,852,671 | 42,701,335 (93.43%) | 41,452,606 (90.70%) | 1,248,729 (2.73%) | 45.62% | 94.27% |
| RP3 | 44,331,630 | 22,165,815 | 41,535,455 (93.69%) | 40,320,789 (90.95%) | 1,214,666 (2.74%) | 45.72% | 94.74% |

*Each sample consists of three biological replicates that are indicated by adding ordinal number suffix to the GP and RP samples.

** The bracketed values represent percentage of the mapped clean reads.


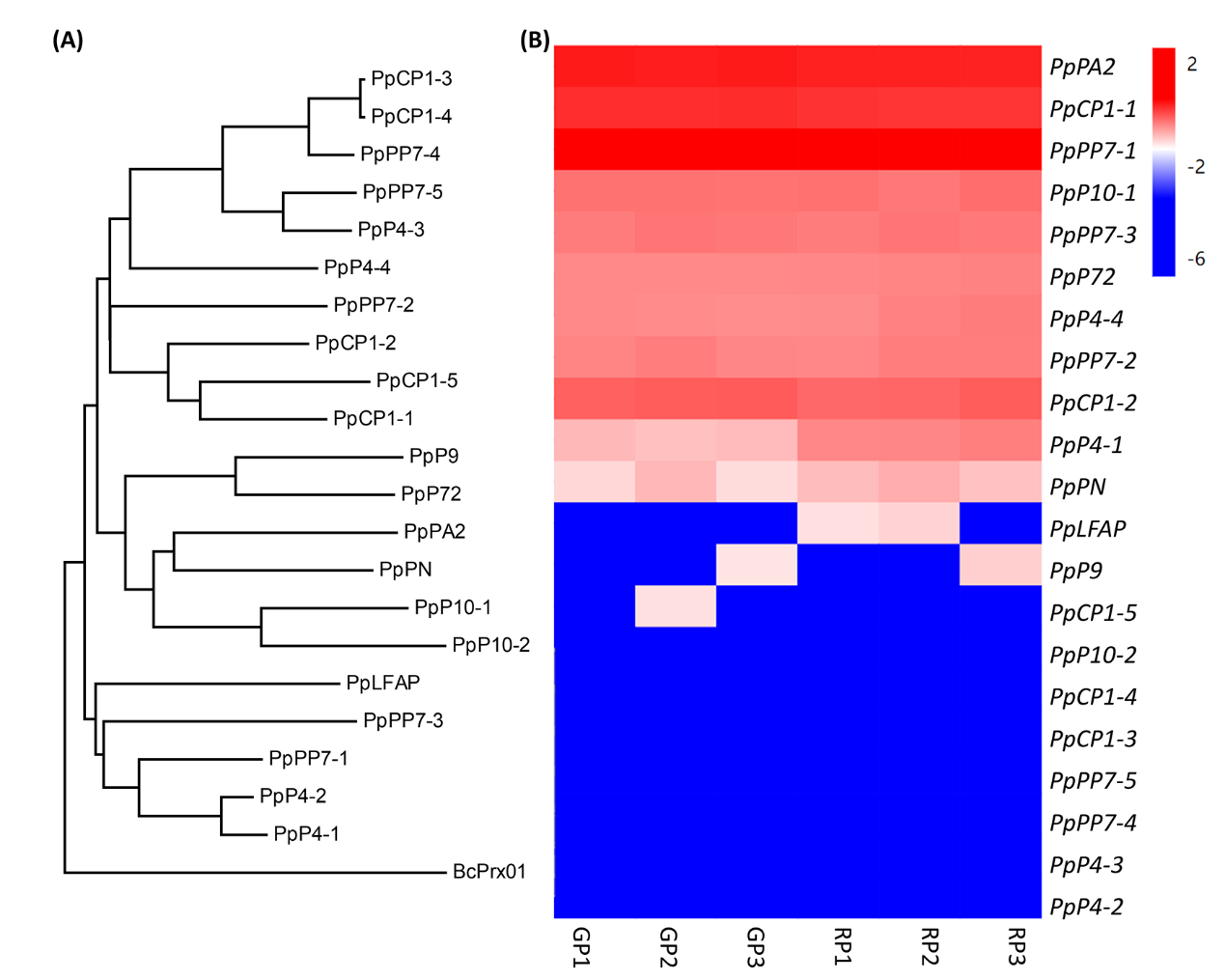


**Fig. S1.** Phylogenetic relationship and gene expression of Peroxidases related to anthocyanin degradation in peach. (A) Phylogenetic tree derived from amino acid sequences of *Pr01s* in peach and orchid. All sequences are retrieved from NCBI database with accessions as follows: *P. persica* PpPP7-1 (XP_007219210.2), PpPP7-2 (XP_007215699.2), PpP4-1 (XP_007205544.1), PpP4-2 (XP_007205551.1), PpCP1-1 (XP_007205557.1), PpCP1-2 (XP_007206416.2), PpP4-3 (XP_007206479.1), PpPP7-3 (XP_007219390.2), PpP4-4 (XP_007218699.2), PpPP7-4 (XP_007206549.2), PpPP7-5 (XP_007206242.1), PpCP1-3 (XP_007207602.1), PpCP1-4 (XP_020420647.1), PpPA2 (XP_007202211.1), PpCP1-5 (XP_007208188.2), PpLFAP (XP_007226601.2), PpPN (XP_007206866.1), PpP9 (XP_007226292.1), PpP10-1 (XP_007202220.1), PpP10-2 (XP_007200388.1), and PpP72 (XP_007202348.1); *B. calycina* BcPr01 (AHB59751.1). (B) Expression of *PpPr01s* in the sun-exposed (RP) and shaded (GP) peel tissues of ‘MLWN’ based on the RNA-seq data.


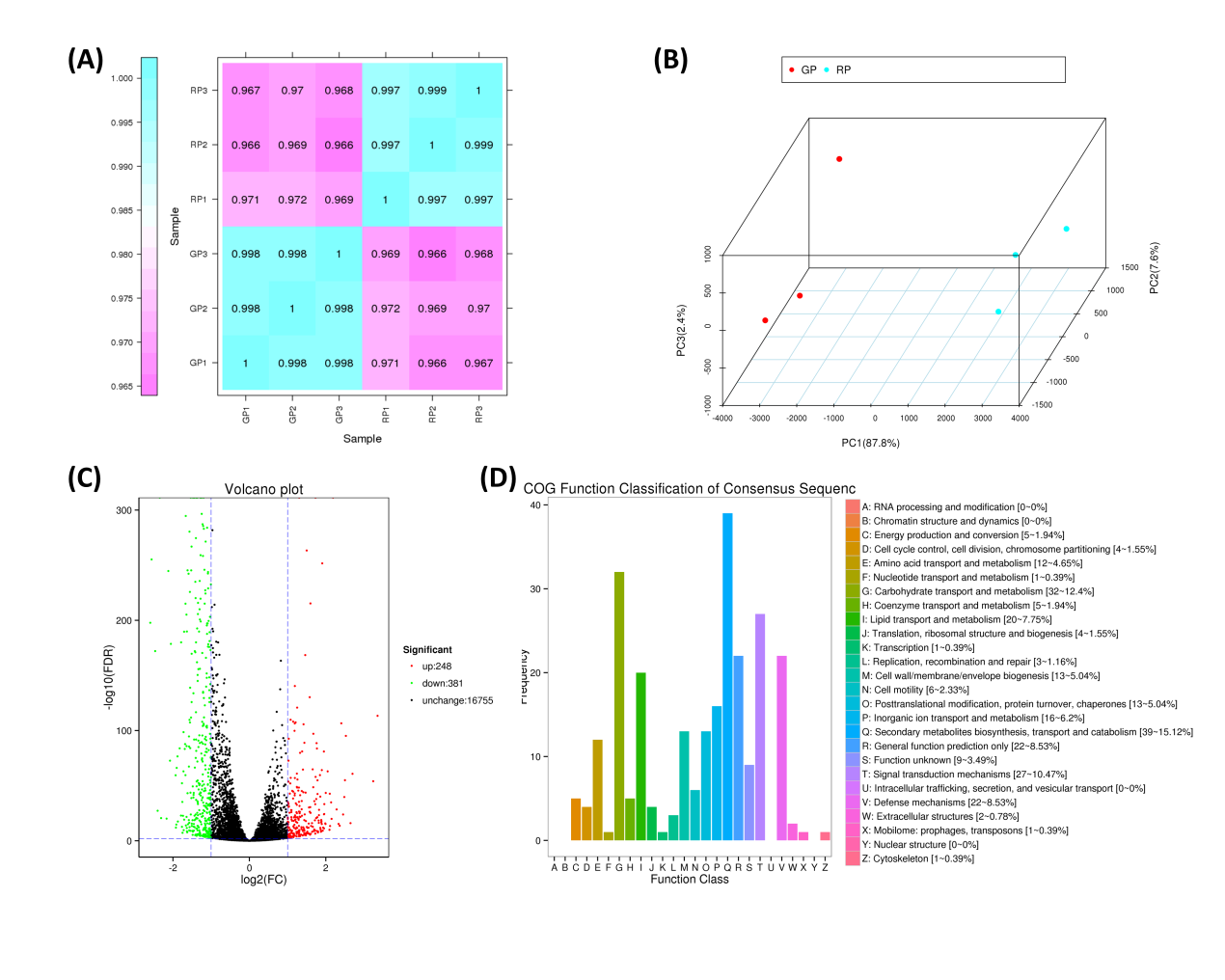


**Fig. S2.** Analysis of transcriptome sequencing data. (A) Correlation analysis between biological replicates. (B) Principal component analysis (PCA) of three biological replicates. (C) Volcano map of DEGs. (D) COG enrichment analysis of DEGs.


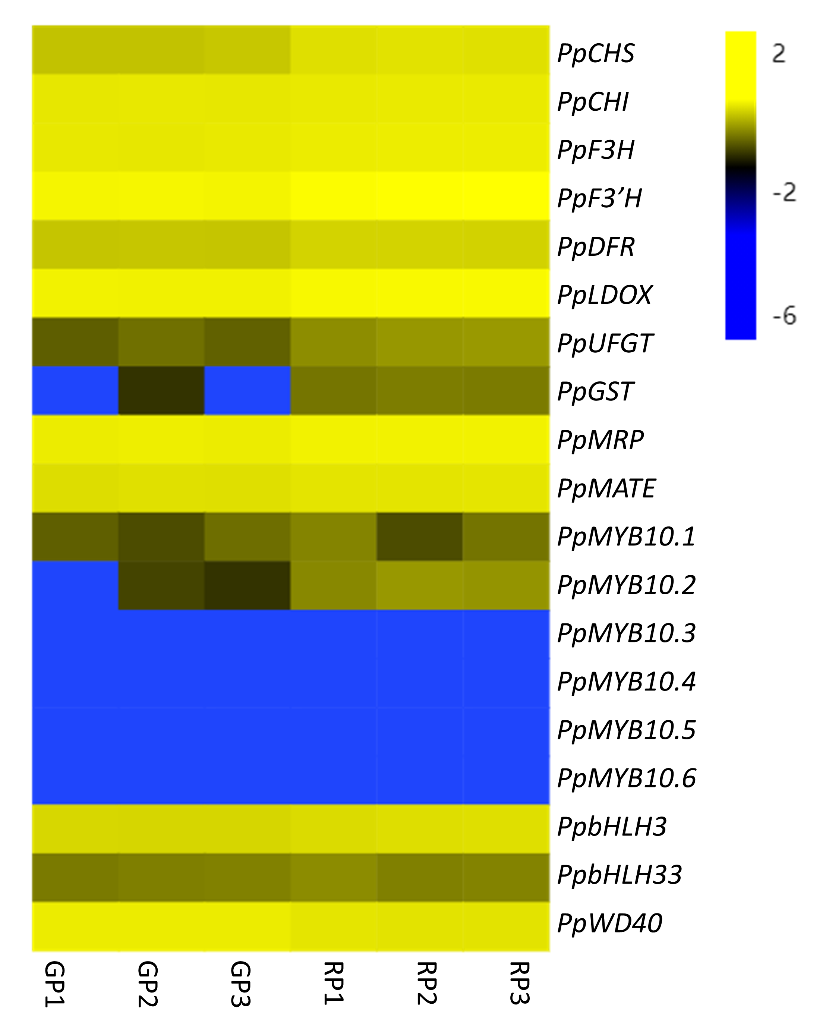


**Fig. S3.** Expression of anthocyanin structural and regulatory genes in the sun-exposed (RP) and shaded (GP) peel tissues of ‘MLWN’ based on the RNA-seq data.


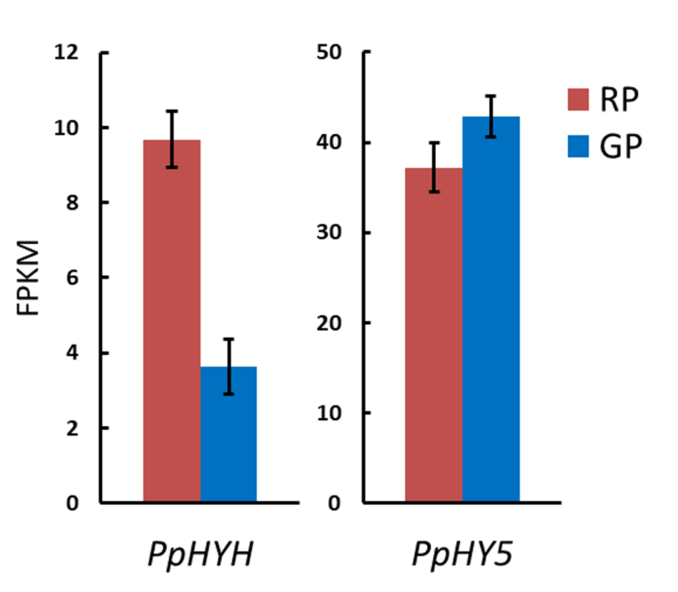


**Fig. S4.** Expression levels of *PpHYH* and *PpHY5* in the sun-exposed and shaded peel tissues of ‘MLWN’ based on the RNA-seq data. The error bar indicates ± SE of three biological replicates.


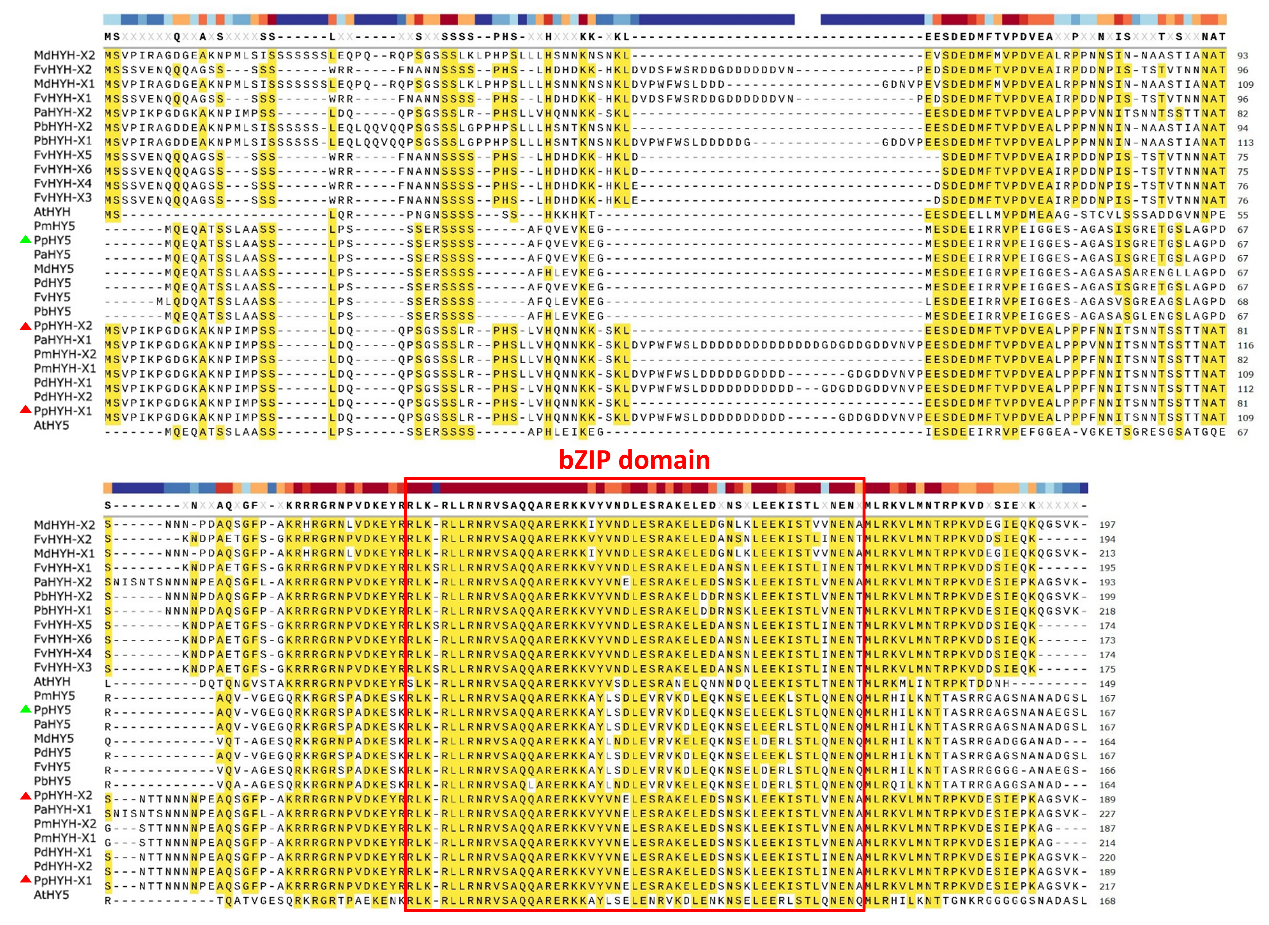


**Fig. S5.** Alignment of amino acid sequences of *HYH* and *HY5* genes in peach and other plant species. The *HYH* and *HY5* genes in peach are highlighted in triangle.


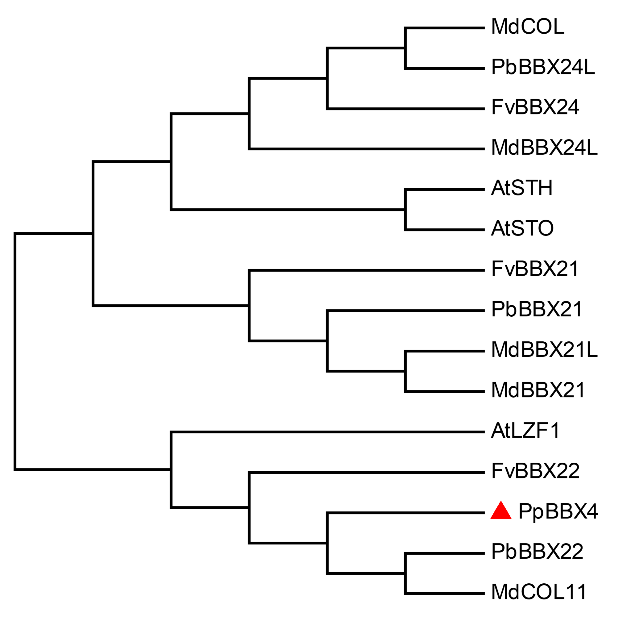


**Fig. S6.** Phylogenetic tree derived from amino acid sequences of *BBX* genes in peach and other plant species. The PpBBX4 gene is highlighted by solid triangles. All sequences are retrieved from NCBI database with accessions as follows: *P. persica* PpBBX4 (XP_007211715.1); *Arabidopsis thaliana* AtLZF1 (NP_565183.1), AtSTO (OAP11786.1) and AtSTH (NP_565722.1); *Fragaria vesca* FvBBX21 (XP_004291815.1), FvBBX22 (XP_004294165.1) and FvBBX24 (XP_004309809.1); *Malus domestica* MdCOL11 (XP_028944947.1), MdBBX21 (XP_008387988.1), MdBBX21L (XP_028950954.1) and MdBBX24L (XP_028956746.1); *Pyrus x bretschneideri* PbBBX21 (XP_009335579.1), PbBBX22 (XP_009376736.1) and PbBBX24L (XP_009342646.1).


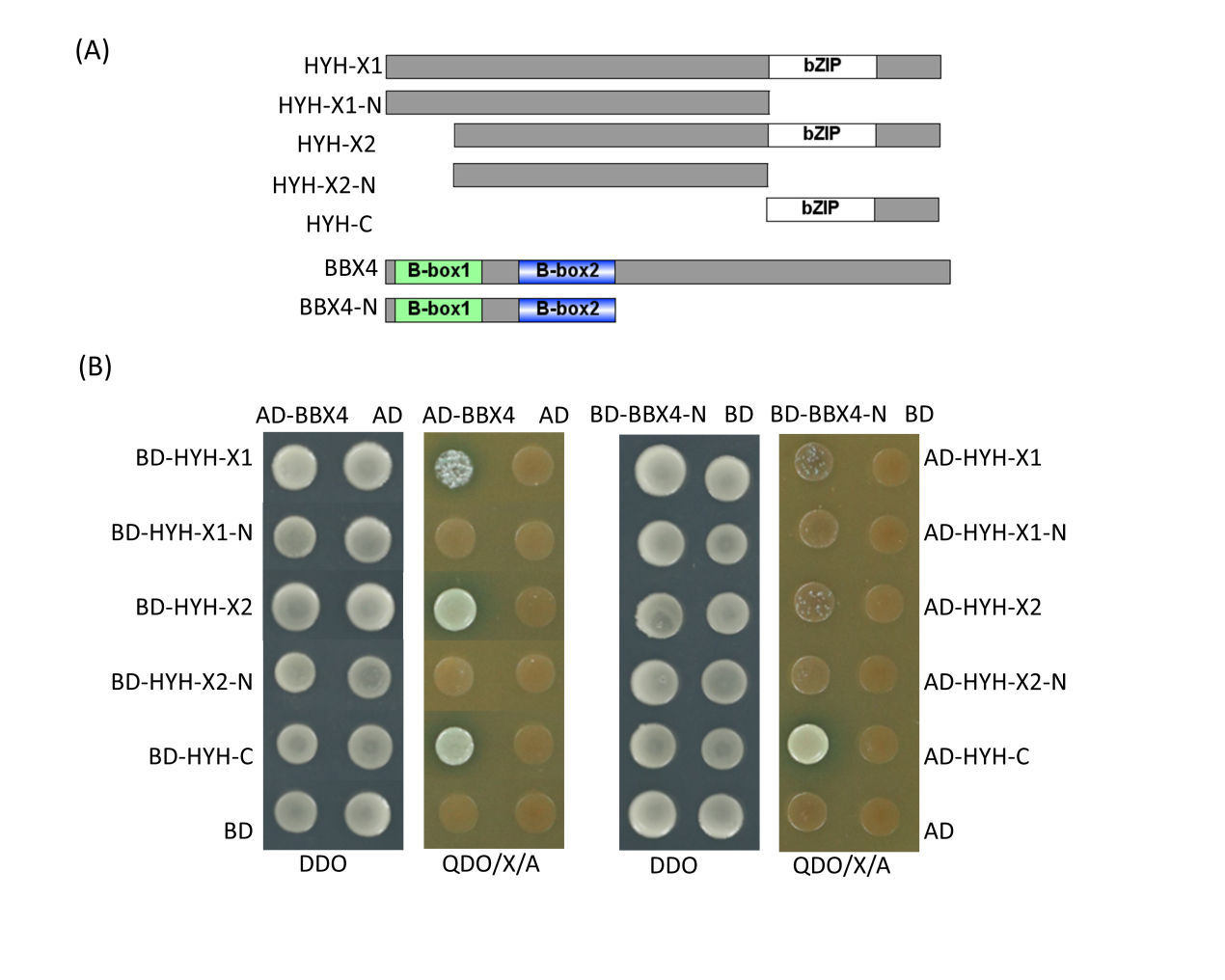


**Fig. S7.** Identification of the key domains responsible for the interaction between PpHYH and PpBBX4. (A) Schematic diagram of protein domain structures of PpHYH and PpBBX4 and their truncated variants. (B) Validation of the interaction between PpHYH and PpBBX4 using Y2H.


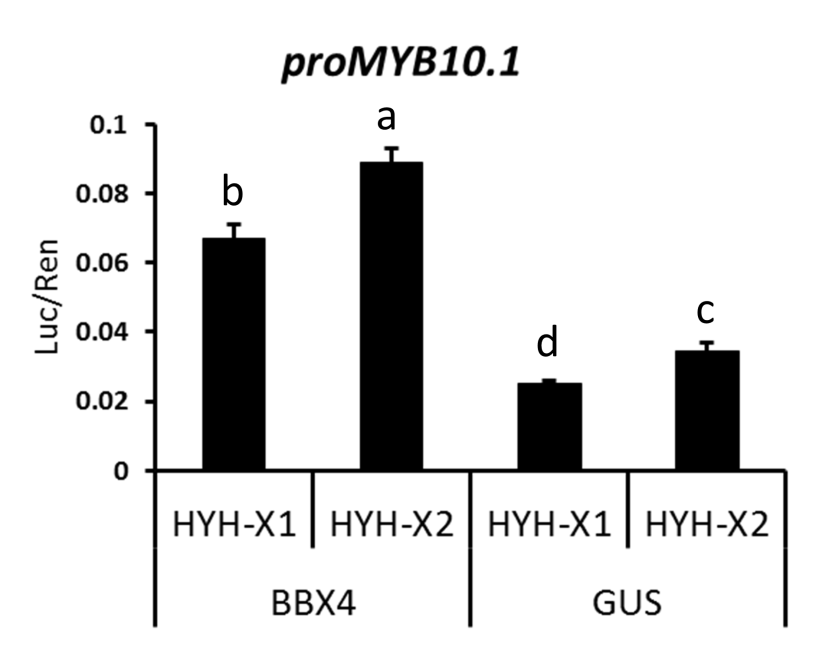


**Fig. S8.** Assessment of the activation of PpHYH on the *PpMYB10.1* promoter using the dual-luciferase reporter system. The error bars indicate ± SE of at least three biological replicates, and significant difference at *P* < 0.05 in is indicted by different lowercase letters based on LSD test.


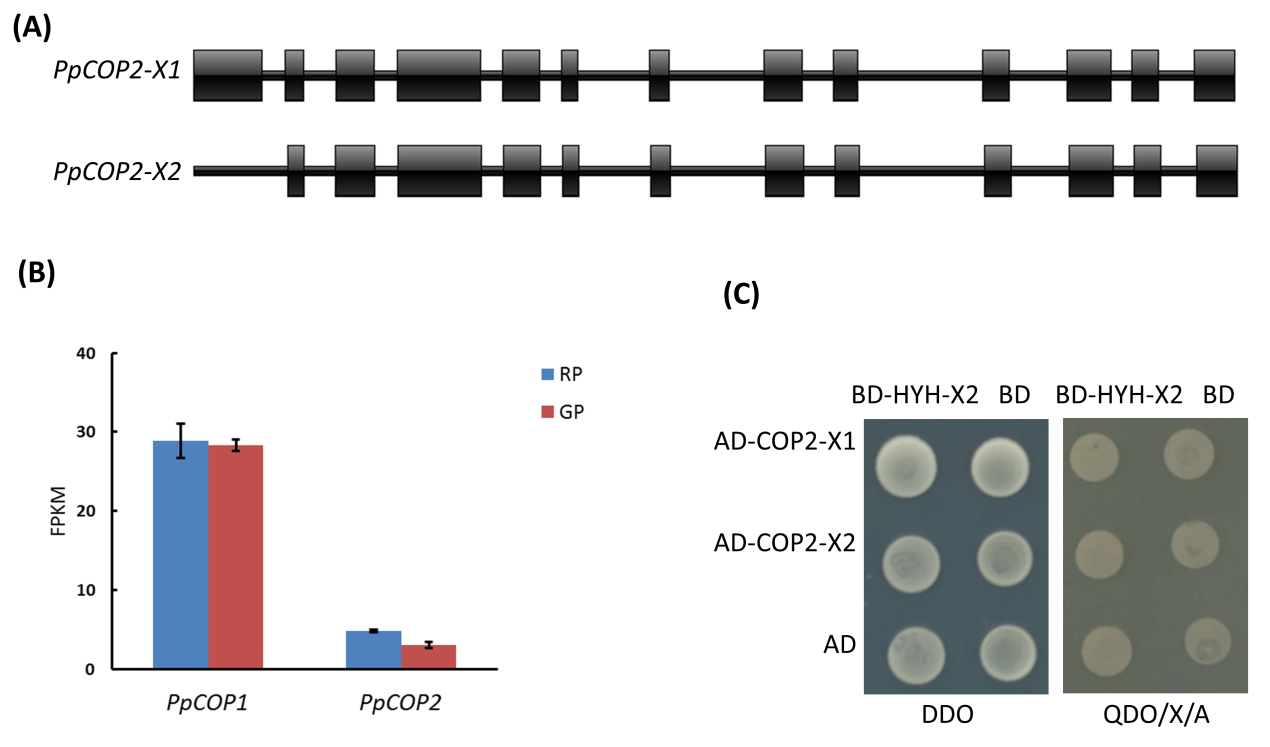


**Fig. S9.** Functional analysis of the *PpCOP2* gene. (A) Two alternative splicing variants of *PpCOP2*. (B) Expression levels of *PpCOP1* and *PpCOP2* in the sun-exposed and shaded peel tissues of ‘MLWN’ based on the RNA-seq data. The error bar indicates ± SE of three biological replicates. (C) Validation of the interaction between PpHYH-X2 and PpCOP2.


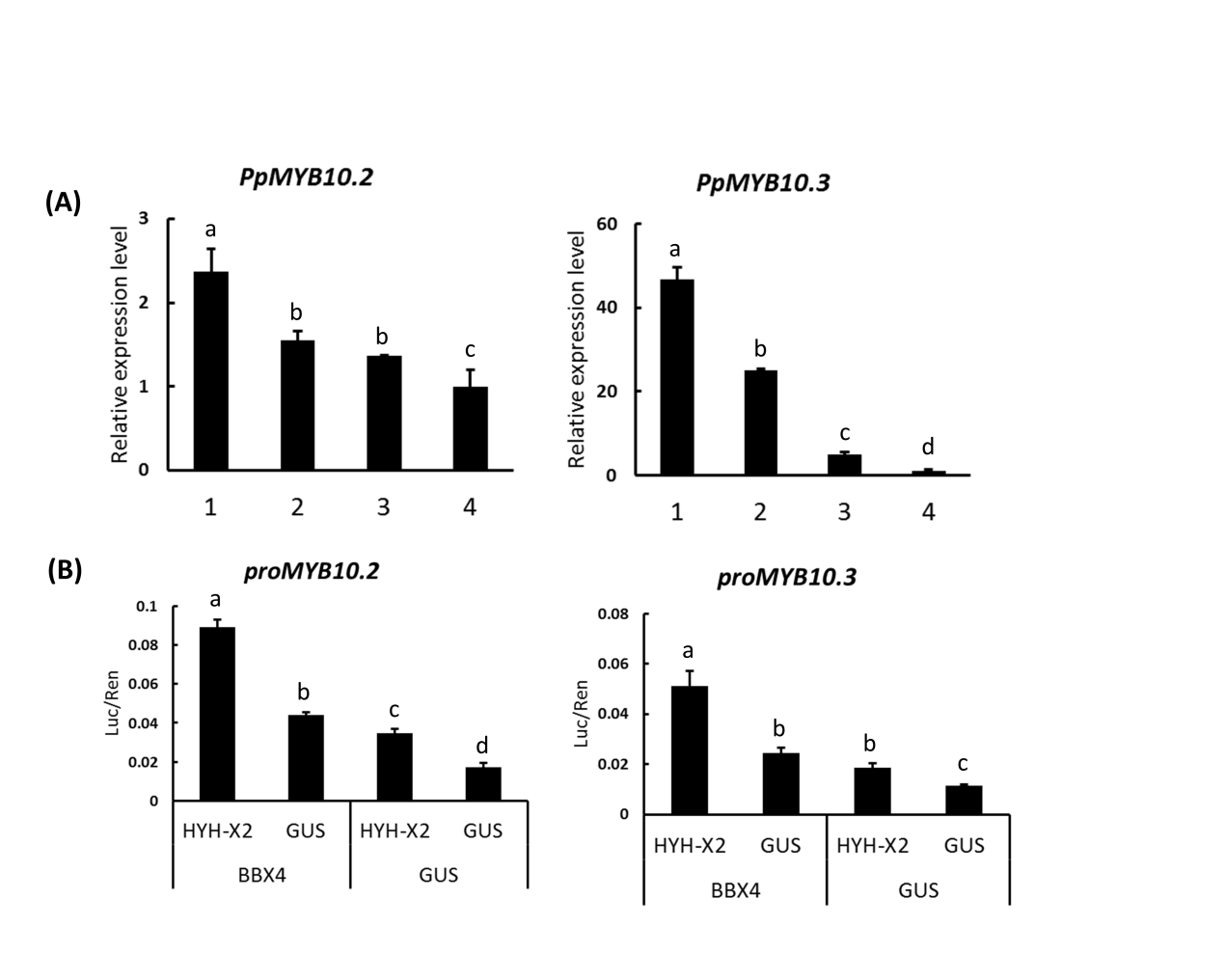


**Fig. S10.** Effect of PpHYH-X2 on anthocyanin-activating MYB genes *PpMYB10.2* and *PpMYB10.3*. (A) Expression of *PpMYB10.2* and *PpMYB10.3* in peach flesh tissues around the same infiltration sites as shown in Figure 4A. (B) Assay of the activation of PpHYH-X2 on the promoters of *PpMYB10.2* and *PpMYB10.3* using the dual-luciferase reporter system. The error bars in (A) and (B) show ± SE of at least three biological replicates, and significant difference at *P* < 0.05 in is indicted by different lowercase letters based on LSD test.


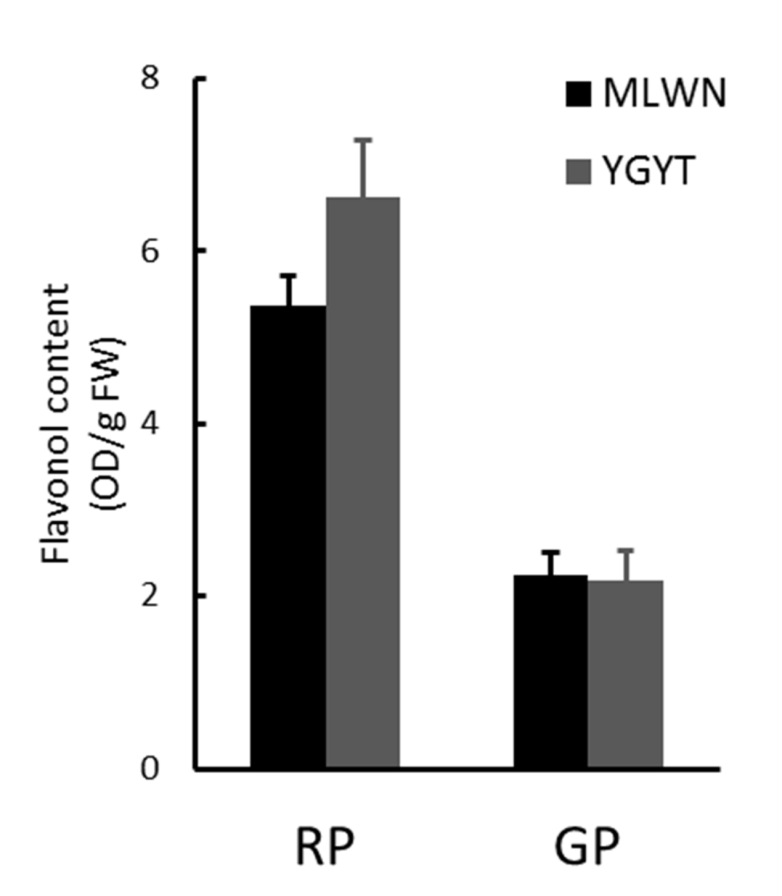


**Fig. S11.** Effect of light on flavonol accumulation in the fruit exocarp of peach cv. MLWN and cv. YGYT.

**
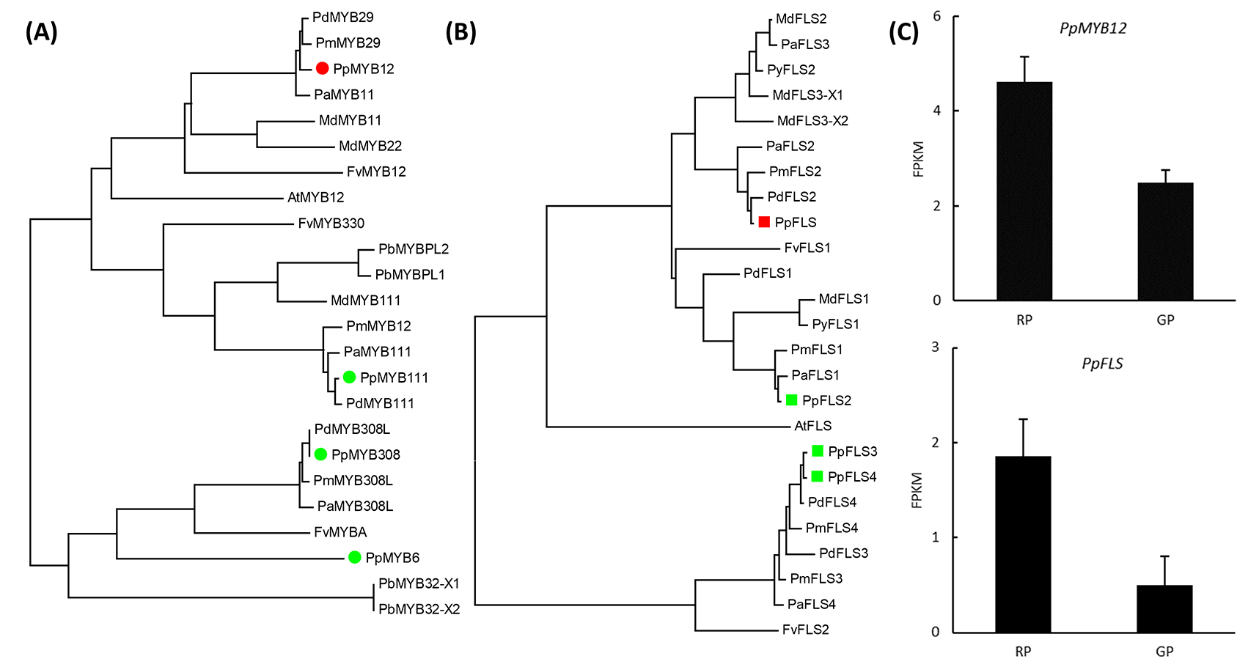
**

**Fig. S12.** Analysis of *MYB12* and *FLS* genes in peach (A) Phylogenetic tree derived from amino acid sequences of *MYB12* gene in peach and other plant species. The genes in peach are highlighted by solid circle. All sequences are retrieved from NCBI database with accessions as follows: *Arabidopsis* AtMYB12 (NP_182268.1); *P. persica* PpMYB12 (XP_020425532), PpMYB111 (XP_020410856), PpMYB308 (XP_007218760) and PpMYB6 (XP_020419855.1); *P. avium* PaMYB11 (XP_021805128), PaMYB111 (XP_021822276) and PaMYB308L (XP_021815015); *Fragaria vesca* FvMYB330 (XP_004295109.1), FvMYB12 (XP_004301809.1), and FvMYBA (NP_001295449.1); *Malus domestica* MdMYB22 (AAZ20438.1), MdMYB11 (XP_028945115.1) and MdMYB111 (XP_028951203.1); *Pyrus x bretschneideri* PbMYBPL1 (XP_009347775.1), PbMYBPL2 (XP_009369961.2), PbMYB32-X1(XP_009340433.1) and PbMYB32-X2 (XP_018499327.1); *P. mume* PmMYB29 (XP_008235184.1), PmMYB12 (XP_008223335.1) and PmMYB308L (XP_008233115.1); *P. dulcis* PdMYB29 (XP_034227164.1), PdMYB111 (XP_034227785.1) and PdMYB308L (XP_034202910.1). (B) Phylogenetic tree derived from amino acid sequences of *FLS* gene in peach and other plant species. The genes in peach are highlighted by solid square. All sequences are retrieved from NCBI database with accessions as follows: *Arabidopsis* AtFLS (NP_196481.1); *P. persica* PpFLS (XP_007222535.1), PpFLS2 (XP_020425583.1), PpFLS3 (XP_007222581.1) and PpFLS4 (XP_007222580); *P. avium* PaFLS1 (XP_021810218.1), PaFLS2 (XP_021810872), PaFLS3 (AFO67943.1) and PaFLS4 (XP_021810338.1); *Fragaria vesca* FvFLS1 (XP_004290837.1) and FvFLS2 (XP_011458773.1); *Malus domestica* MdFLS1 (XP_028950966.1), MdFLS2 (NP_001306179.1), MdFLS3-X1 (XP_028962807.1) and MdFLS3-X2 (XP_028962808.1); *Pyrus x bretschneideri* PyFLS1 (XP_009367022.1) and PyFLS2 (XP_009341666.1); *P. mume* PmFLS1 (XP_008233897.1), PmFLS2 (XP_008233898.1), PmFLS3 (XP_008233895.1) and PmFLS4 (XP_008226888.1); *P. dulcis* PdFLS1 (BBG95663.1), PdFLS2 (XP_034217337.1), PdFLS3 (XP_034198176.1) and PdFLS4 (XP_034198457.1). (C) The FPKM values of *PpFLS* and *PpMYB12* in the sun-exposed (RP) and shaded (GP) peel tissues of ‘MLWN’. The error bars indicate ± SE of three biological replicates.
